# The R2R3-type MYB transcription factor *MdMYB90-like* is responsible for the enhanced skin color of an apple bud sport mutant

**DOI:** 10.1038/s41438-021-00590-3

**Published:** 2021-07-01

**Authors:** Chao Sun, Chunming Wang, Wang Zhang, Shuai Liu, Weiyao Wang, Xinyi Yu, Tao Song, Maxwell Yu, Weichang Yu, Shenchun Qu

**Affiliations:** 1 College of Horticulture, Nanjing Agricultural University, No. 1 Weigang, 8210095 Nanjing, China; 2 Guangdong Key Laboratory of Plant Epigenetics, College of Life Sciences and Oceanography, Shenzhen University, 518060 Shenzhen, China; 3 Shenzhen Research Institute, The Chinese University of Hong Kong, Shenzhen, China; 4 Department of Biomedical Engineering, Washington University in St.Louis, St.Louis, MO 63130 USA

**Keywords:** Transcriptional regulatory elements, Secondary metabolism

## Abstract

The anthocyanin content in apple skin determines its red coloration, as seen in a Fuji apple mutant. Comparative RNA-seq analysis was performed to determine differentially expressed genes at different fruit development stages between the wild-type and the skin color mutant. A novel R2R3-MYB transcription factor, MdMYB90-like, was uncovered as the key regulatory gene for enhanced coloration in the mutant. The expression of MdMYB90-like was 21.3 times higher in the mutant. *MdMYB90-like* regulates anthocyanin biosynthesis directly through the activation of anthocyanin biosynthesis genes and indirectly through the activation of other transcription factors that activate anthocyanin biosynthesis. *MdMYB90-like* bound to the promoters of both structural genes (*MdCHS* and *MdUFGT*) and other transcription factor genes (*MdMYB1* and *MdbHLH3*) in the yeast one-hybrid system, electrophoretic mobility shift assay, and dual-luciferase assay. Transgenic analysis showed that MdMYB90-like was localized in the nucleus, and its overexpression induced the expression of other anthocyanin-related genes, including *MdCHS*, *MdCHI*, *MdANS*, *MdUFGT*, *MdbHLH3*, and *MdMYB1*. The mutant had reduced levels of DNA methylation in two regions (−1183 to −988 and −2018 to −1778) of the *MdMYB90-like* gene promoter, which might explain the enhanced expression of the gene and the increased anthocyanin content in the mutant apple skin.

## Introduction

A bud sport is a naturally occurring mutation in a tree branch, and these have been widely used by breeders to select desirable characteristics for breeding^1,2^. The phenotypes of bud sport mutants mainly include early development, fruiting spurs, and coloring. In recent years, high-throughput sequencing technology has been widely used in studies to uncover the molecular mechanisms of these natural mutations. In one Fuji apple bud mutant, SNPs (single-nucleotide polymorphisms) and unique InDels (insertions or deletions) were detected by using whole-genome resequencing^3^. In a “Yanfu 6” apple spur-type mutant, microRNAs played important roles in regulating the shoot apical meristem, cell division, and internode length^4^. MYB transcription factors and epigenetic regulation were reported in an anthocyanin-deficient yellow-skinned somatic mutant ‘Blondee’^5^. In European pear, the methylation level of *PcMYB10* is associated with the formation of green-skinned sports^6^. A large number of excellent varieties have been produced by utilizing bud mutants in fruit trees, such as apple, pear, cherry, orange, and grape^4,7–11^.

Anthocyanins are secondary metabolites that have antioxidant and antitumor functions as well as activity against coronary heart disease and even help to defend against pathogens and ultraviolet radiation^12–14^. Apple fruit coloration not only determines fruit appearance and economic characteristics but also has beneficial value for human health. Therefore, it is considered an important trait for apple breeding^15,16^. Both structural genes and regulatory genes in the anthocyanin pathway have been proven to be important for fruit skin color. *MdPAL* (phenylalanine ammonia lyase), *MdCHS* (chalcone synthase), *MdCHI* (chalcone isomerase), *MdF3H* (flavanone 3-hydroxylase), *MdDFR* (dihydroflavonol 4-reductase), *MdANS* (anthocyanidin synthase), and *MdUFGT* (UDP-glucose flavonoid 3-O-glucosyltransferase) have been discovered to have positive correlations with the accumulation of anthocyanin in apple skin^17–21^. Moreover, regulatory genes affect fruit skin color through the regulation of structural genes^22^. Ectopic expression of apple MdMYB1 can activate both *DFR* and *UFGT* structural genes involved in anthocyanin biosynthesis in tobacco^23^. MdMYBA can bind specifically to an anthocyanidin synthase (*MdANS*) promoter region to regulate anthocyanin synthesis in apple skin^24^.

In recent years, multiple omics technologies, such as mRNA sequencing, miRNA sequencing, metabolomic, and proteomic analyses, have been used in the analysis of apple mutants^5,25–27^. A color mutant of a Fuji apple with early coloring and red skin pigmentation was discovered in Jiangsu Province, China^25^. Proteomics investigations identified 451 differentially expressed proteins in the fruit skin of this mutant. The mutant had significantly increased expression of photosynthesis-related proteins, stress-related proteins, and proteins in the anthocyanin biosynthesis pathway, but the expression of mitogen-activated protein kinase 4 (MAPK4) and mevalonate kinase (MVK) was substantially downregulated, indicating posttranscriptional regulation of skin color formation in the mutant. To understand the transcriptional regulation of anthocyanin biosynthesis in this mutant, comparative RNA-seq analysis of a Fuji apple and its color mutant was performed, a key regulatory gene, *MdMYB90-like*, was uncovered as a novel R2R3-type MYB transcription factor, and the mechanism of its regulation of anthocyanin biosynthesis was discussed.

## Results

### M_Fuji apple mutant skin color development occurred earlier than that of wild-type Fuji

**Fig. 1:**
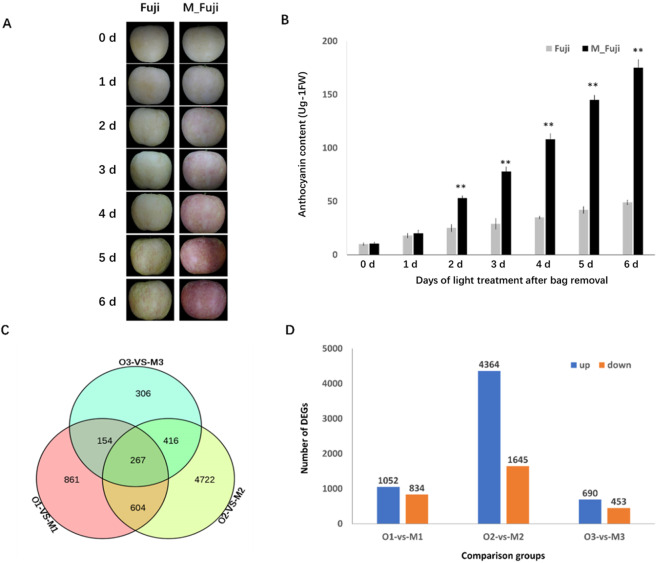
Differentially expressed genes (DEGs) between the Fuji apple and the mutant. **A** Fruits of Fuji and M_Fuji were exposed to different durations of light treatment after bag removal. **B** Anthocyanin contents in skins of Fuji and M_Fuji fruits. Samples were assayed on light-treated days after bag removal. Error bars are SEs for three replicates. **C** Venn diagram of unique and common DEGs at three different stages. **D** Number of DEGs upregulated (blue) or downregulated (orange) at three different stages (O1-VS-M1, O2-VS-M2, O3-VS-M3)

Mature apple fruits from the skin color mutant (M_Fuji) had redder skin than the original yellowish-green skin with a red flush (Fig. 1A). Skin color development was light-dependent with little anthocyanin when bagged but attained the highest level at 6 days after bag removal (DABR) under continuous light treatment (Fig. 1B). In comparison, the anthocyanin content increased slowly in the wild-type Fuji apples. A significant difference was observed between the mutant and the wild-type apple at 2 DABR under continuous light treatment (Fig. 1A, B).

### Transcriptome analysis of M_Fuji mutant and wild-type Fuji apples during fruit development

Skin samples from wild-type Fuji (O) and M_Fuji (M) apples were collected at 4, 8, and 12 DABR. Six cDNA libraries representing the 6 treatments (O1, M1, O2, M2, O3, M3) were constructed with total RNA and subjected to Illumina deep sequencing. The number of clean reads ranged from 20,209,566 in the O3 group to 27,419,664 in the O2 group. High-quality reads represented 98.41–99.38% of the total clean reads with a Q30>91% and N% <0.00 in all groups, indicating that the transcriptome data were of high quality (Supplementary Table S1).

Comparative analysis of the RNA-seq data identified 1886, 6009, and 1143 DEGs between wild-type Fuji and the mutant at three different stages (O1-vs-M1, O2-VS-M2, O3-VS-M3) (Supplementary Tables S2–S4), of which 267 DEGs were consistently observed in all three comparison groups (Fig. 1C). A total of 1052 upregulated DEGs and 834 downregulated DEGs were obtained between the O1 and M1 libraries, 4364 upregulated genes, and 1645 downregulated genes were identified between the O2 and M2 libraries, and 690 upregulated DEGs and 453 downregulated DEGs were found between the O3 and M3 libraries. Upregulated DEGs outnumbered downregulated DEGs in the M_Fuji group at all three stages (Fig. 1D).

Genes involved in the anthocyanin biosynthesis pathway among the 267 DEGs shared by the three developmental stages were analyzed. These genes included six structural genes and two MYB transcription factors. Among the six structural genes, DEGs encoding phenylalanine ammonia lyase (PAL, LOC103433222, LOC103430265), 4-coumarate: coenzyme ligase (4CL, LOC103426517), chalcone synthase (CHS, LOC103443512, LOC103443513), chalcone isomerase (CHI, LOC103430446), anthocyanin synthase (ANS, LOC103437326, LOC103437327) and UDP-glucose flavonoid 3-O-glucosyltransferase (UFGT, LOC103440008, LOC103420802) were all upregulated in the mutant.

Structural genes in anthocyanin biosynthesis are largely regulated at the transcriptional level by the MYB–bHLH-WD40 protein complex^28^. The protein complex is composed of three types of transcription factors: the R2R3-MYB transcription factor, the basic helix–loop–helix (bHLH) transcription factor, and the WD40 protein. Among the differentially expressed MYB transcription factors, MdMYB1 (LOC103444202) and an unknown transcript, Tcons_00045044 were consistently upregulated in the mutant. MdMYB1 was the first identified R2R3-type MYB transcription factor regulating anthocyanin biosynthesis in apple^29^. Transcript Tcons_00045044 showed high homology with the *PbMYB90-like* of pear and was thus designated *MdMYB90-like*. In addition, a *MdbHLH3* (LOC103449015) transcription factor was upregulated at stage 2, but no WD40 genes were detected among the DEGs.

### Quantitative real-time PCR (qRT-PCR) validation of DEGs

**Fig. 2:**
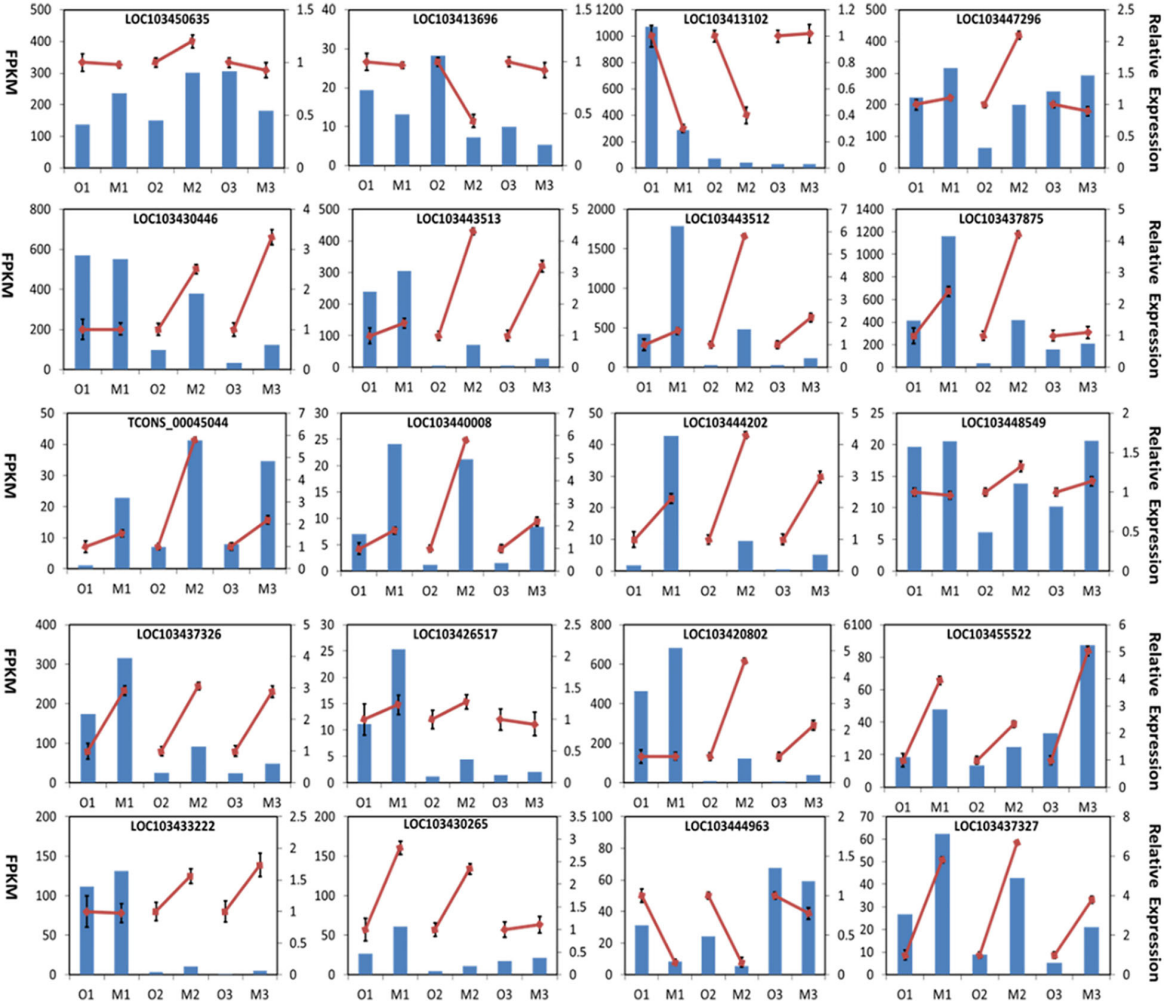
RNA-seq and qRT-PCR results of 20 selected DEGs in Fuji and mutant apples. The left *y* axis indicates the corresponding expression data from RNA-seq (blue histogram). The right *y* axis shows the relative gene expression level measured by qRT-PCR (red lines). Bars represent the standard error (SE; *n* = 3)

Twenty selected candidate DEGs were analyzed by qRT-PCR to validate the transcriptomic data and to profile their expression during the apple coloration process. Among these genes, 12 structural genes, including two upregulated *PAL*s (LOC103433222, LOC103430265), one upregulated *4CL* (LOC103426517), one upregulated *4-coumarate-CoA ligase-like* (LOC103447296), two *CHS*s (LOC103443512, LOC103443513), one *CHI* (LOC103430446), one upregulated *DFR* (LOC103448549), two upregulated *ANS*s (LOC103437326, LOC103437327), two upregulated *UFGT*s (LOC103440008, LOC103420802), and two MYB transcription factors, including *MdMYB90-like* (HF36881-RA) and *MdMYB1* (LOC103444202), were verified as involved in the anthocyanin biosynthesis pathway. In addition, genes in the flavonoid biosynthesis pathway, including one downregulated flavonol synthase gene (*FLS*, LOC103413102), one upregulated *flavonoid 3’-monooxygenase F3’H* (LOC103437875), one downregulated *anthocyanidin reductase ANR* (LOC103413696), and one upregulated *anthocyanidin-3-o-glucosyltransferase 5-like* (LOC103455522), were confirmed. Two upregulated *DMR6-like oxygenase 1 s* (LOC103450635, LOC103444963) were also verified by qRT-PCR (Fig. 2).

### Isolation and analysis of the MdMYB90-like transcription factor

Transcript (Tcons_00045044) was extremely upregulated during the three-phase periods in the mutant (Fig. 2). When its sequence was BLASTed against the apple genome (*Malus×domestica* HFTH1 V1.0 a1 transcripts), it best matched the gene HF36881-RA. The HF36881-RA gene showed high homology with *PbMYB90-like* of pears in the NCBI GenBank database and thus was designated *MdMYB90-like*. The coding sequence (CDS) of *MdMYB90-like* was 621 bp and encoded a putative protein of 206 amino acids with an ATG start codon at nucleotide position 1 and a TGA stop codon at position 3297 (Supplementary Fig. S1A). Both *PbMYB90-like* and *MdMYB90-like* had similar gene structures, including three exons and two introns, and the first two exons were 130-bp long. Furthermore, the R2 domain of *MdMYB90-like* consisted of exon 1 and part of exon 2, while the R3 domain was split over exons 2 and 3.

The MdMYB90-like protein structure was consistent with that for other previously reported R2R3 MYBs^30^. Phylogenetic relationships among plant R2R3-type MYB transcription factors, including MdMYB90-like, *Arabidopsis* MYB transcription factors, and anthocyanin-related MYBs of Rosaceae were constructed by neighbor-joining methods (Supplementary Fig. S1B). Interestingly, MdMYB90-like clustered with multiple previously verified anthocyanin-related MYB transcription factors in fruit trees, including MdMYB1, MdMYB10, MdMYBA, and PbMYB10 (Supplementary Fig. S1B). Protein sequence alignment of MdMYB90-like, MdMYB1 and previously reported MYB transcription factors, including *Arabidopsis* AtMYB113, AtMYB114, AtMYB75, AtMYB90*, Pyrus bretschneideri* PbMYB10, PbMYB90-like, *Fragaria ananasa* FaMYB1, and *Malus domestica* MdMYB10, MdMYB3, and MdMYBA, revealed that these MYB TFs were conserved in both R2 and R3 DNA-binding domains in the N-terminal region (Supplementary Fig. S1C). However, more diversity was found in the C-terminal region. In addition, all but the MdSIMYB1 proteins had the previously reported R/B-like bHLH motif ([D/E] Lx2[R/K]x3 Lx6 Lx3R) in the R3-DNA domain^31^. Protein sequence alignment indicated that the amino acid identity between MdMYB1 and MdMYB90-like was 68.44%. Two anthocyanin-related conserved motifs, M1 motif [A/S/G]N[D/A/N]V and M2 motif ([K/R] Pxxx[K/T] [F/Y]), were identified in MdMYB90-like. While the C-terminal M2 motif [RPQPQKF] was identical for MdMYB90-like and MdMYB1, the M1 motif [A/S/G]N[D/A/N]V in MdMYB90-like (Ser–Asn–Asp–Val) was different from that in MdMYB1 (Ala–Asn–Ala–Val) (Supplementary Fig. S1C).

### Subcellular localization of the MdMYB90-like protein

To determine the subcellular location of *MdMYB90-like*, the full-length CDSs of *MdMYB90-like* were inserted into the pC29_35S:GFP5_his6 vector. An empty 35S:GFP vector was used as the negative control, while MdMYB1 was used as the positive control. The constructs were transformed into onion epidermal cells by biolistic transformation and into tobacco leaves by agroinfiltration. GFP fluorescence was observed in the MdMYB1-GFP- and MdMYB90-like-GFP-transformed onion and tobacco nuclei, while fluorescent signals were observed in both the nucleus and cytoplasm of the empty 35S:GFP vector (Supplementary Fig. S2). These results indicated that, similar to MdMYB1, MdMYB90-like proteins were also localized in the nucleus.

### Analysis of cis-elements in gene promoters

To further characterize the function of this transcription factor, a 2018-bp region upstream of the translation start site in the *MdMYB90-like* gene (the putative promoter sequence) was cloned and analyzed through the PlantCARE program. Multiple MYB-binding elements, light-responsive elements (G-boxes), GT1 motifs, and several hormone-responsive elements, such as abscisic acid-responsive elements, auxin-responsive elements, and MeJA-responsive elements, were detected (Supplementary Fig. S3). These results suggested that the expression of the *MdMYB90-like* gene might be regulated by various factors, such as abscisic acid, jasmonic acid, gibberellin, and light. Mitosis-specific activator (MSA)-like elements were found in the *MdMYB90-like* gene promoter, which indicated that it might be involved in cell cycle regulation.

In addition, the promoter regions of *MdMYB1*, *MdCHS*, *MdUFGT*, *MdANS*, and *MdbHLH3* were also isolated and analyzed by PlantCARE. Cis-elements, including hormone-responsive elements, light-responsive elements, low-temperature elements, and MYB-binding elements, were found in MdMYB1. MYB-binding elements, light-responsive elements, and several hormone-responsive elements were found in the promoters of *MdCHS*, *MdUFGT*, *MdANS*, and *MdbHLH3*, such as abscisic acid-responsive elements and MeJA-responsive elements (Supplementary Fig. S3).

### Functional analysis of MdMYB90-like by overexpression in transgenic materials

**Fig. 3:**
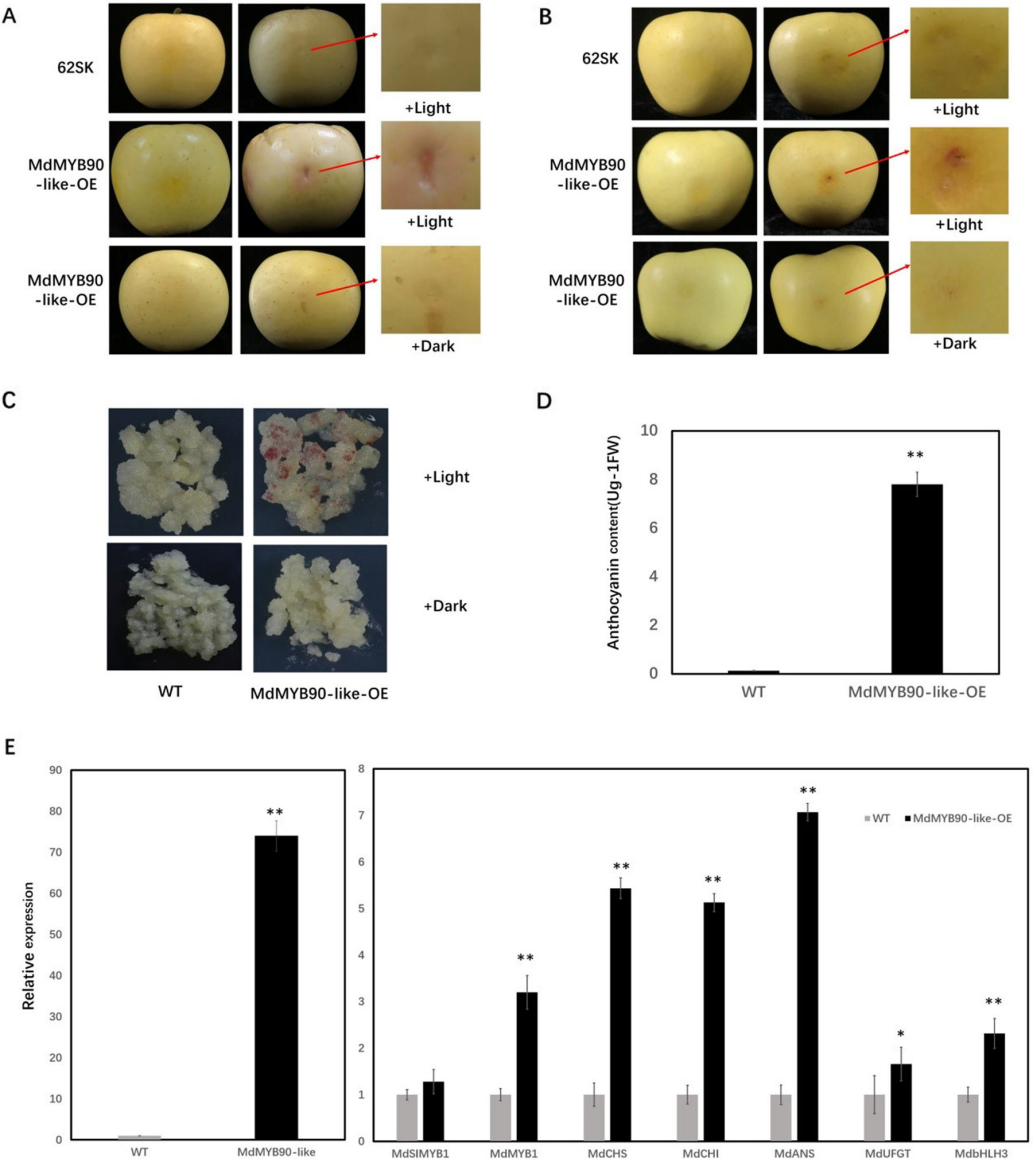
Anthocyanin biosynthesis and gene expression in apple transgenic lines. **A**, **B** Anthocyanin accumulation in agroinfiltrated Fuji (**A**) and Golden Delicious (**B**) apple skin after 3 and 5 days of treatment, respectively. Agrobacterium harboring the MdMYB90-like overexpression vector and the 62SK empty vector were infiltrated into apple skins and exposed to light and dark, respectively. **C** Accumulation of anthocyanin in MdMYB90-like overexpression calli (MdMYB90-like-OE) and wild-type calli (WT) after 5 days of light and dark treatment. WT calli were used as the control. **D** Anthocyanin contents of transgenic (MdMYB90-like-OE) and wild-type calli (WT). **E** Expression levels of *MdMYB90-like*, *MdSIMYB1*, *MdMYB1*, *MdCHS*, *MdCHI*, *MdANS*, and *MdUFGT* in transgenic and wild-type apple calli. Asterisks (*) and (**) denote significant differences between samples at *P* < 0.05 and *P* < 0.01 significance levels, respectively

To study the biological function of MdMYB90 in the regulation of anthocyanin biosynthesis, an overexpression vector (62SK-MdMYB90-like) was constructed and transformed into apple skins by transient agroinfiltration in both Fuji and Golden Delicious apple. Anthocyanin accumulated when 62SK-MdMYB90-like was transformed and cultured under continuous light for 3–5 days; however, no anthocyanin accumulation was observed when the empty vector 62SK was transformed or when 62SK-MdMYB90-like transformed apple was cultured in the dark, indicating the requirement of light for anthocyanin biosynthesis. Anthocyanin accumulation was faster in Fuji (3 days) than Golden Delicious (5 days) apple (Fig. 3A, B and Supplementary Fig. S4).

The function of *MdMYB90-like* was also studied in stably transformed apple calli by the agrobacterium-mediated transformation of “Orin” apple calli. Transgenic calli that overexpressed the *MdMYB90-like* gene under the 35S promoter began to show red spots 2 days after transfer to light conditions (Fig. 3C), but no visible changes were observed in wild-type calli or transgenic calli cultured in the dark. Light-dependent anthocyanin biosynthesis was again confirmed in the transgenic calli. The anthocyanin content was analyzed 5 days after light exposure and was found to be significantly higher in transgenic *MdMYB90-like* calli than in the WT control (Fig. 3D).

Gene expression was analyzed in transgenic calli (Supplementary Fig. S4). The expression of the *MdMYB90-like* gene was more than 80 times higher in transgenic calli than in WT calli. In addition, the overexpression of the *MdMYB90-like* gene promoted the expression of both structural (*MdCHS*, *MdCHI*, *MdANS*, and *MdUFGT*) and regulatory genes (*MdMYB1* and *MdbHLH3*) in the anthocyanin biosynthesis pathway (Fig. 3E). In contrast, the transcription level of the unrelated gene *MdSIMYB1* was not induced by MdMYB90-like overexpression in apple calli (Fig. 3E). These results suggested that *MdMYB90-like* might promote apple anthocyanin accumulation directly by activating anthocyanin biosynthesis genes and indirectly by activating other transcription factors.

### Transcriptional activity of MdMYB90-like

**Fig. 4:**
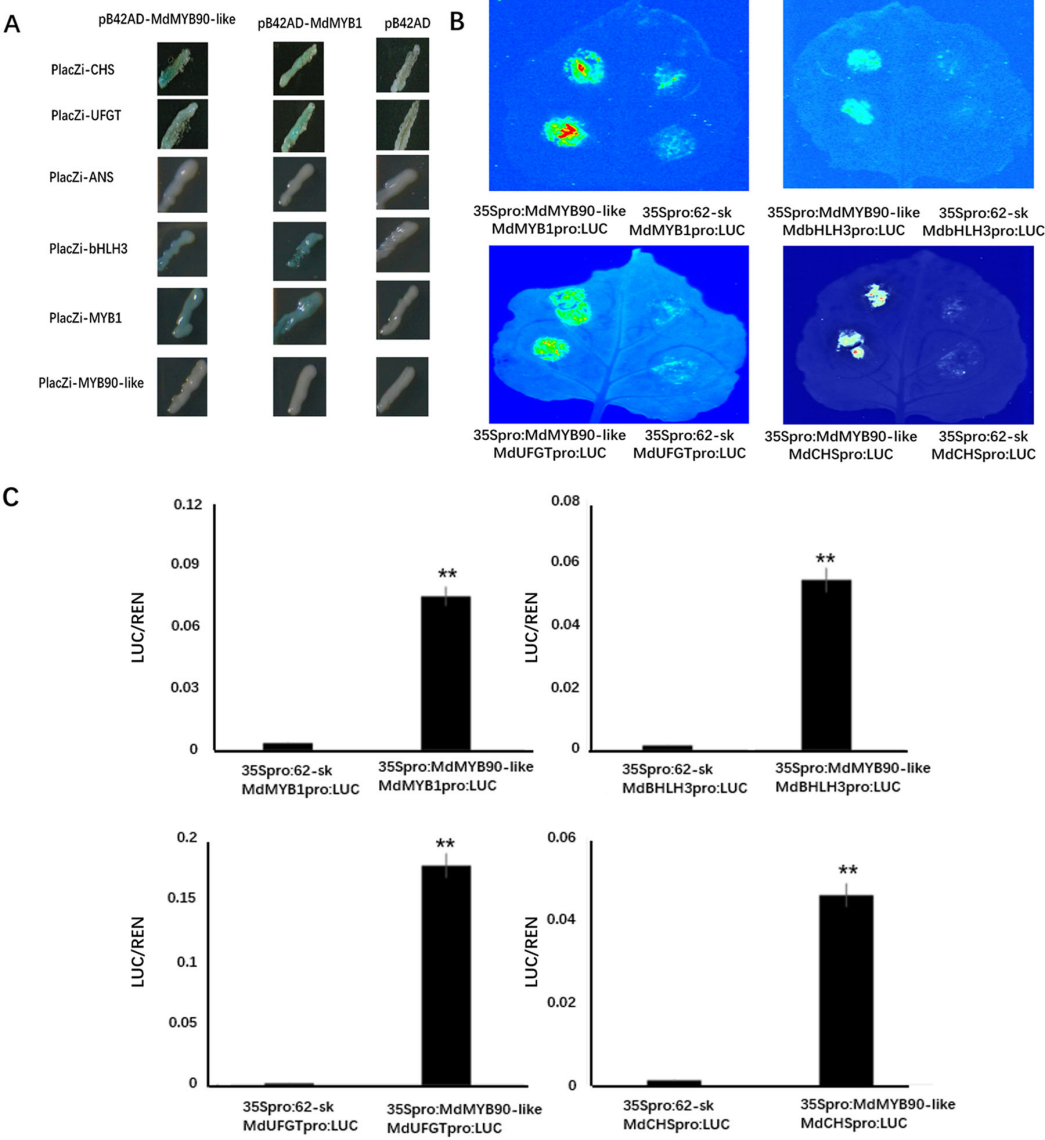
Transcriptional activity of MdMYB90-like. **A** Yeast one-hybrid (Y1H) analysis of interactions of MdMYB90-like (left panels) and MdMYB1 (middle panels) with the promoters of anthocyanin biosynthesis-related genes. The pB42AD vector was used as the negative control (right panels). **B** Dual-luciferase detection experiments showed that MdMYB90-like promoted the expression of the *MdCHS*, *MdBHLH3*, *MdUFGT*, and *MdMYB1* genes. **C** LUC/REN activities of constructs: 35Spro:MdMYB90-like/MdMYB1pro:LUC and 35Spro:62-SK/MdMYB1pro:LUC; 35Spro:MdMYB90-like/MdbHLH3pro:LUC and 35Spro:62-SK-MdbHLH3pro:LUC; 35Spro:MdMYB90-like/MdUFGTpro:LUC and 35Spro:62-SK-MdUFGTpro:LUC; 35Spro:MdMYB90-like/MdCHSpro:LUC and 35Spro:62-SK-MdCHSpro:LUC

MdMYB1 activates the expression of downstream anthocyanin biosynthesis genes by interacting with MYB cis-elements in their promoters^23^. MYB-binding elements (MBS, CAACTG) were detected in the promoters of *MdCHS*, *MdUFGT*, *MdANS* and *MdbHLH3*, *MdMYB1*, and *MdMYB90-like* genes. To determine whether MdMYB90-like could interact with these genes, a yeast one-hybrid (Y1H) assay was performed. The results showed that MdMYB90-like could bind to the promoters of *CHS* and *UFGT* genes. MdMYB90-like also bound to the *MdMYB1* and *MdbHLH3* promoters but not to its own promoter. In contrast, MdMYB1 could bind to its own promoter as well as promoters of other genes (*CHS*, *UFGT*, and *MdBHLH3*) but not to the *MdMYB90-like* promoter (Fig. 4A). These results suggested possible regulation of both structural and regulatory genes by MdMYB90-like.

Direct binding of MdMYB90-like protein to MYB-binding elements (MBS) in the *MdCHS*, *MdUFGT*, *MdMYB1*, and *MdBHLH3* promoters was revealed by an electrophoretic mobility shift assay (EMSA). MdMYB90-like protein bound to all probes containing the MBS elements from different gene promoters. The binding could be reduced by competitors containing the MBS elements but not reduced by the MBS mutants, indicating that MdMYB90-like could specifically recognize these MBS elements in the promoters (Supplementary Fig. S5).

To further analyze the activation of anthocyanin biosynthesis genes by MdMYB90-like, MdMYB90-like was cotransformed into tobacco leaves with constructs containing the promoters of *MdCHS*, *MdUFGT*, *MdbHLH3*, and *MdMYB1* fused to the firefly *LUC* gene (Fig. 4B). The results showed activation of LUC activity in all cotransformations with MdMYB90-like (Fig. 4C), demonstrating the regulatory activity of MdMYB90-like on these anthocyanin biosynthesis genes.

#### Mechanism of *MdMYB90-like* and *MdMYB1* gene upregulation in the apple mutant

Sequence variations in the promoter region of an apple mutant have been reported to contribute to the differential expression of regulatory genes and fruit color^33^. To determine whether sequence variations existed in our mutant, *MdMYB90-like* and *MdMYB1* gene sequences from Fuji and its mutant were analyzed. The 621-bp coding region and a 2018-bp region upstream of the *MdMYB90-like* gene were cloned from both the Fuji apple and the mutant and sequenced. No sequence difference was detected in the gene-coding region, while only two single-nucleotide polymorphisms (SNPs) were found in the promoter region at −1293 bp and −634 bp upstream of the translation start codon. Similarly, no sequence difference was found in either the promoter or CDS of *MdMYB1* between the Fuji apple and the mutant.

**Fig. 5:**
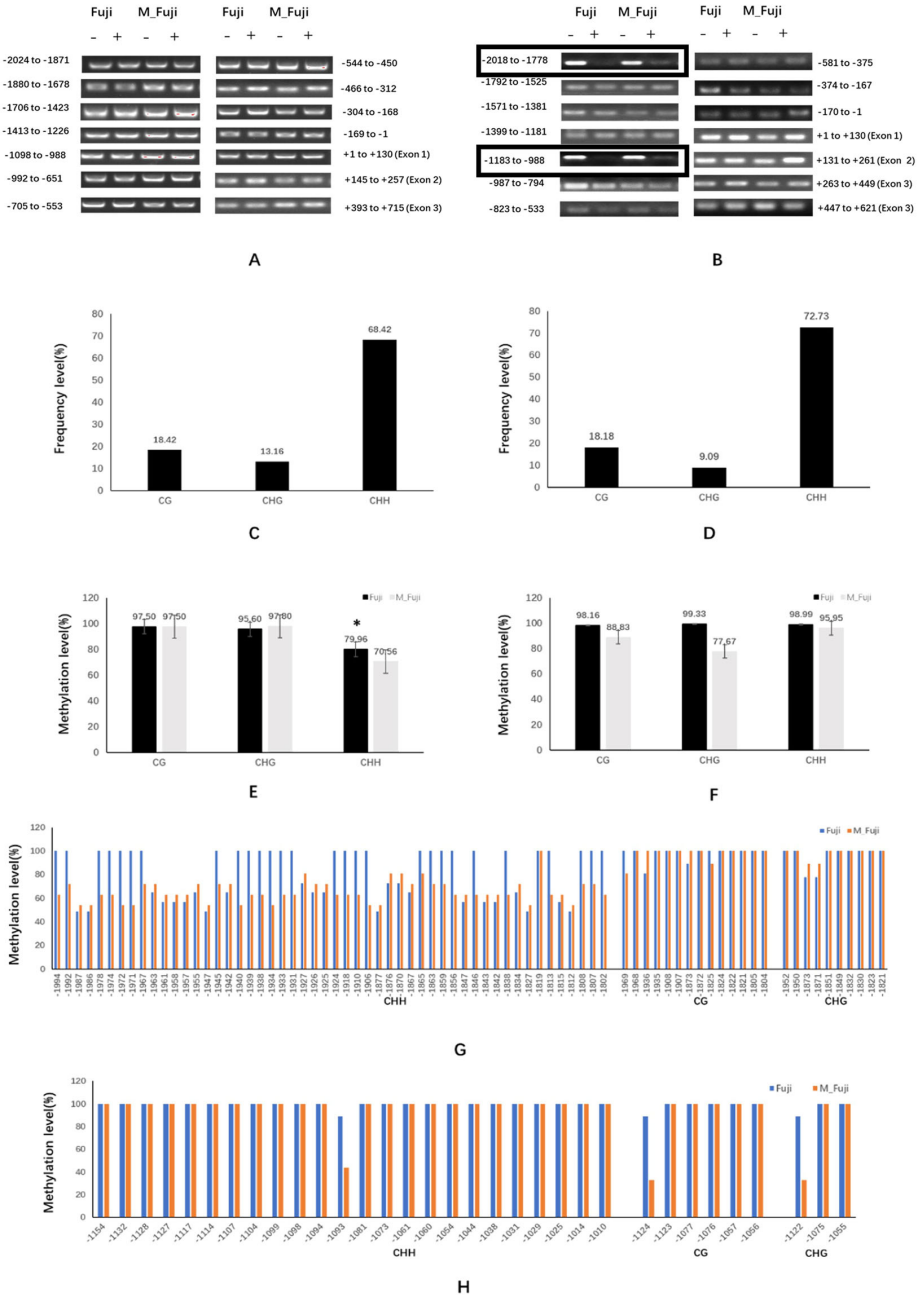
DNA methylation analysis by McrBC-PCR and bisulfite sequencing. **A**, **B** DNA methylation analysis by McrBC-PCR. Genomic DNAs from both Fuji and mutant (M_Fuji) apple skins were treated with McrBC digestion reactions with (+) or without (−) GTP. The promoters and CDSs of MdMYB1 (**A**) and MdMYB90-like (**B**) of both Fuji and the mutant (M_Fuji) were divided into fourteen regions, and each fragment was PCR-amplified. The numbers denote the start and end positions of each fragment relative to the “A” nucleotide (+1) of the translation initiation codon. **C**–**H** DNA methylation analysis by bisulfite sequencing. Types of cytosine methylation sites (CG, CHG, and CHH) in the −1997 to −1800 (**C**) and −1162 to −1009 (**D**) regions of the MdMYB90-like promoter; methylation levels in the −1997 to −1800 (**E**) and −1162 to −1009 (**F**) regions in MdMYB90-like promoters of both Fuji and mutant (M_Fuji) apple skins; methylation levels of individual cytosine across the two regions: −1997 to −1800 (**G**) and −1162 to −1009 (**H**) in both Fuji and the mutant (M_Fuji). The methylation level at each cytosine position represented the average of nine sequenced bisulfite-PCR clones. Three biological replications were performed, and the means and SEs of methylation levels (percentage of methylated nucleotides) were calculated. Asterisks (*) denote significant differences at the *P* < 0.05 level

DNA methylation has been reported as another reason for the regulation of gene expression in apple mutants^34^. To analyze DNA methylation in the promoters and the CDSs of the *MdMYB1* and *MdMYB90-like* genes, McrBC-PCR analysis was conducted. McrBC is an endonuclease that cleaves DNA containing methylcytosine on one or both DNA strands. For the *MdMYB1* gene, the promoter and the gene-coding regions of both the mutant and the normal Fuji apple showed low levels of methylation because all fragments were resistant to McrBC digestion, and no visible difference was detected after PCR amplification (Fig. 5A). However, differences were detected in two regions of the *MdMYB90-like* promoters (−1183 to −988 and −2018 to −1778) (Fig. 5B). DNA methylation levels in these two regions were high because they were sensitive to McrBC digestion. When we compared the normal Fuji apple and the mutant, we found evidence of relatively higher methylation in the normal Fuji apple because the fragments almost completely disappeared after McrBC digestion, while a weak band could be recognized in the mutant (Fig. 5B).

To confirm these results, bisulfite sequencing (BSP)-PCR was performed to detect cytosine methylation in the two regions of the *MdMYB90-like* promoter in the Fuji apple and the mutant. In the −2018 to −1778 region, 76 cytosine positions, including 52 CHH (68.42%), 14 CG (18.42%), and 10 CHG (13.16%) types of cytosine methylation sites, were detected in a 198-bp DNA fragment (−1997 to −1800) (Fig. 5C, G). High levels of methylation were detected in both the Fuji apple and the mutant; however, the methylation level in the Fuji apple (79.96%) was significantly higher than that in the mutant (70.56%) in the CHH-type cytosines (Fig. 5E, G). Similarly, in the −1183 to −988 region, 33 cytosine positions, including 24 CHH (80%), 6 CG (18.18%), and 3 CHG (9.09%) types of cytosine methylation sites, were detected in a 154-bp DNA fragment (−1162 to −1009) (Fig. 5D, H). The overall methylation levels were high in both the Fuji apple and the mutant. Reduced methylation was detected in the mutant at three positions: CG at −1124, CHG at −1122, and CHH at −1093 (Fig. 5F, H). The bisulfite sequencing data were in good agreement with those of the McrBC-PCR analysis and confirmed the reduced methylation in the mutant in the two promoter regions.

## Discussion

### Candidate genes for skin color mutation

Apple color reflects the anthocyanin content in the apple skin. In red delicious apples, the red mutant showed earlier coloration, and a higher anthocyanin content correlated with higher expression of genes related to anthocyanin biosynthesis^35^. The anthocyanin biosynthesis pathway has been well-studied in various plants^36^. It begins with 4-coumaroyl-coenzyme A (CoA), a metabolic intermediate from the phenylpropanoid pathway. The synthesis of naringenin chalcone from 4-coumaroyl-coenzyme A is the first commitment step for anthocyanin biosynthesis and is catalyzed by chalcone synthase (CHS). In this study, various genes in the anthocyanin biosynthesis pathway were detected as differentially expressed genes between Fuji apples and the mutant. These genes include two *PAL* genes (LOC103433222, LOC103430265) and one *4CL* gene (LOC103426517) in the phenylpropanoid pathway, three *CHS* genes (LOC103443512, LOC103421794, LOC103443513), one *CHI* gene (LOC103430446), one *DFR* gene (LOC103448549), two *ANS* genes (LOC103437326, LOC103437327), and three *UFGT* genes (LOC103417897, LOC103420802, LOC103428842). PAL and 4CL work in the phenylpropanoid metabolism pathway to produce 4-coumaroyl-coenzyme A from phenylalanine. Studies found that the expression of *PAL* was positively correlated with the synthesis of anthocyanin in strawberries and apples^37,38^. The increased expression of two *PAL* genes in this study was also positively correlated with the increased anthocyanin content in the apple mutant (Fig. 2). CHS is the key enzyme in anthocyanin biosynthesis. Three *CHS* transcripts (LOC103443512, LOC103421794, LOC103443513) were shown to be greatly upregulated in the mutant. Interestingly, these three *CHS* transcripts were also detected in Granny Smith apples during fruit development, and their activation by 5-aza-20-deoxycytidine (5-aza-dC) treatment enhanced apple coloration^39^. Silencing of *CHS* in transgenic Royal Gala apple significantly reduced the anthocyanin content^40^. CHI catalyzes the conversion of chalcone to flavanones. We observed the activated expression of a *CHI* (LOC103430446) in the mutant. The same gene was shown to be activated in Granny Smith apples after 5-aza-20-deoxycytidine treatment, and the anthocyanin content was upregulated^39^. The upregulated ANS (LOC103437326, LOC103437327) plays a role in the oxidation of colorless anthocyanidins to produce colored anthocyanins. UFGT catalyzes the glycosylation of anthocyanidins to anthocyanins and has been found to contribute to cyanidin 3-galactoside biosynthesis in apple skin^41^. Three *UFGT* genes (LOC103417897, LOC103420802, and LOC103428842) were upregulated in our apple mutant (Fig. 2), and they were also upregulated in Granny Smith apples after 5-aza-dC treatment, which promoted anthocyanin accumulation^39^. *UFGT* was also reported to be the key gene determining white or red grape phenotypes^42^. The increased expression of proteins in the anthocyanin biosynthesis pathway was also reported in the same mutant by proteomics^25^. The coordinately induced genes in the anthocyanin biosynthesis pathway in the apple mutant might suggest the involvement of transcription factors, which have been reported to directly regulate the expression of structural genes^43^. Indeed, we observed differentially expressed transcripts encoding MYB and bHLH transcription factors. A new apple MYB transcription factor, MdMYB90-like, was characterized in detail and was found to be the key regulatory gene for enhanced anthocyanin biosynthesis in the mutant.

### MdMYB90-like is the key regulator in apple anthocyanin biosynthesis

The two-repeat (R2R3) MYB family is the largest family characterized in plants. A large number of these proteins have been isolated and proven to regulate anthocyanin biosynthesis in many plant species^44^. In apples, *MdMYB1*, *MdMYB10*, and *MdMYBA* were identified to be responsible for anthocyanin accumulation by regulating the expression of anthocyanin biosynthesis structural genes. For example, previous studies have shown that *MdMYB10* mainly enhances anthocyanin content in apples by upregulating the expression of the *DFR* gene^47^. *MdMYB1* can activate both *DFR* and *UFGT* structural genes to regulate anthocyanin biosynthesis, and *MdMYBA* can bind specifically to an anthocyanidin synthase (*MdANS*) promoter region to regulate anthocyanin synthesis in apple skin^23,24^.

RNA-seq data analysis detected two obviously upregulated R2R3-type MYB transcription factors in the apple color mutant. MdMYB1 has been shown to be involved in the regulation of anthocyanin biosynthesis in apple skin^29^. It has been reported that different methylation statuses in the *MdMYB1* promoter region affect its expression and subsequently regulate anthocyanin biosynthesis in the Ralls apple mutant^34^. However, our results showed no difference in either the nucleotide sequence or methylation level in either the coding region or promoter between Fuji and the mutant. Thus, the increased *MdMYB1* expression was more likely to be affected by factors other than the cause of the mutant phenotype.

A novel transcript that encoded a basic R2R3-MYB transcription factor was identified for its consistent upregulation in the mutant at all three stages. Its protein sequence had the highest homology with the pear PbMYB90-like protein and thus was designated MdMYB90-like. MdMYB90-like formed a cluster with the anthocyanin-related MYB transcription factors MdMYB1, MdMYBA and MdMYB10 in apples^23,24,47^. Two conserved motifs, M1 [A/S/G]N[D/A/N]V in the R2R3 domain and M2 [R/K]Px [P/A/R]xx [F/Y] in the C-terminus for anthocyanin-promoting MYBs^46^, were identified in both MdMYB90-like and MdMYB1 (Supplementary Fig. S1C). While the C-terminal motif [RPQPQKF] was identical for MdMYB90-like and MdMYB1, the [A/S/G]N[D/A/N]V motif in the R2R3 domain was different. The different motif sequences in MdMYB90-like (Ser–Asn–Asp–Val) might differentiate it from MdMYB1 (Ala–Asn–Ala–Val) for gene regulation.

MYB transcription factors have been reported to interact with bHLH transcription and form a complex with bHLH to regulate anthocyanin biosynthesis^45^. MdMYB1 has been reported to interact with MdbHLH3 in apples to form a MYB–bHLH complex^32^. MdMYB90-like also contained the bHLH interaction motif [D/E] Lx2[R/K]x3 Lx6 Lx3R in the R3 domain, indicating its potential to interact with the bHLH partner. Compared with MdMYB1, MdMYB90-like had a difference of only three amino acids in the bHLH motif sequence. Our results showed that *MdbHLH3* expression could be induced by MdMYB90-like in transgenic apple calli and that MdMYB90-like could bind to the promoter of *MdbHLH3* in the YIH assay (Fig. 4A). In addition, MdMYB90-like could activate the expression of MdbHLH3 in the dual-luciferase assay (Fig. 4B, C), indicating that MdMYB90-like could be another partner with *MdbHLH3* in apples.

Analysis of the cis-acting element in the promoter regions showed multiple cis-elements in structural genes (*MdCHS*, *MdUFGT,* and *MdANS*) as well as regulatory genes (*MdbHLH3*, *MdMYB1*, and *MdMYB90-like*), including MYB-binding elements (MBS), light-responsive elements (G-box, ACE, GT1-motif, and TCCC-motif), hormone-responsive elements (ABRE for abscisic acid response, CGTCA-motif for MeJA response, and GARE motif for gibberellin response), and elements for low-temperature (LTR) and cell cycle (MSA-like) responses (Supplementary Fig. S3). This explains the regulation of anthocyanin biosynthesis by various environmental and genetic factors and the light dependence of anthocyanin biosynthesis (Figs. 1 and 3).

YIH assay, EMSA, and dual-luciferase assay showed that MdMYB90-like could bind and activate the expression of both structural genes (*MdCHS* and *MdUFGT*) and regulatory genes (*MdbHLH3* and *MdMYB1*) (Fig. 4), which had MYB-binding elements in their promoters. One exception was the *MdANS* gene, which showed activation in transgenic apple calli but was not activated in the Y1H assay. Transgenic apple calli that overexpressed MdMYB90-like also activated the expression of these genes (Fig. 3C–E), demonstrating the regulatory role of MdMYB90-like in apples. Overexpression of MdMYB90-like either in a transient assay in apple skins or in stably transformed apple calli resulted in the accumulation of anthocyanin under light conditions (Fig. 3).

MdMYB1 also bound to both structural genes (*MdCHS* and *MdUFGT*) and regulatory genes (*MdbHLH3* and *MdMYB1*) in the Y1H assay (Fig. 4A). MdMYB1 activated its own expression in Y1H cells, indicating the possibility of self-activation in apples. Although MYB-binding elements were also present in the *MdMYB90-like* promoter, the Y1H assay showed no activation of *MdMYB90-like* by either MdMYB1 or itself (Fig. 4A). The interaction and activation of *DFR* and *UFGT* genes by *MdMYB1* were reported in transgenic tobacco^23^, while MdMYBA was found to bind specifically to the *MdANS* promoter region for its activation^24^.

Our results indicated that MdMYB90-like activated anthocyanin biosynthesis in the apple mutant by both direct activation of anthocyanin biosynthesis genes (*MdCHS* and *MdUFGT*) and indirect activation of these genes through other transcription factors (*MdMYB1* and *MdbHLH3*). This might explain the activation of *MdANS* in transgenic apple calli. Although MdMYB90-like could not activate *MdANS* directly, it could activate *MdMYB1*. An identical protein of MdMYB1, MdMYBA, was reported to directly interact with the *MdANS* promoter and activate its transcription^24^. As an important regulator of anthocyanin biosynthesis in apple, the expression of MdMYB1 is affected by many environmental factors and plant hormones^23^^,29,48–50^ and regulated by other genes^48–50^. For example, *MdEIL1* was found to directly bind to the promoter of MdMYB1 and transcriptionally activate its expression during ethylene-regulated fruit ripening and anthocyanin accumulation^50^. The regulatory role of *MdEIL1* is similar to that of *MdMYB90-like*. Two other genes (*MdTCP46* and *MdBT2*) were reported to regulate MdMYB1 expression in light-induced anthocyanin biosynthesis^49^. MdTCP46 binds to the MdMYB1 protein and promotes its transcriptional activity, while MdBT2 ubiquitinates and degrades the MdTCP46 and MdMYB1 proteins under low-light conditions.

### Methylation of the MdMYB90-like promoter may be the reason for different skin pigmentation patterns

DNA methylation or demethylation in gene promoters affects gene expression, and the expression of MYB transcription factors can have an impact on anthocyanin accumulation in apple skin^34,51^. In this study, neither significant sequence variation nor changes in DNA methylation levels were detected in *MdMYB1* between the Fuji apple and the mutant. However, significant changes in DNA methylation were found in two regions of the *MdMYB90-like* promoters. Unlike the other regions of the *MdMYB90-like* promoter that had low levels of methylation (Fig. 5), two regions (−1183 to −988 bp and −2018 to −1778 bp) were hypermethylated, but differences in methylation levels were recognized between the Fuji apple and the mutant. The mutant showed significantly lower levels of methylation in the two regions (Fig. 5). The lower levels of methylation may explain the increased expression of the *MdMYB90-like* gene, the upregulation of other anthocyanin biosynthesis genes and their regulators, and the enhanced fruit color. Similar results were reported in Gala apples, in which the methylation levels in the *MdMYB10* promoter were negatively correlated with anthocyanin contents in the yellow-skinned somatic mutant Blondee and its red-skinned parent Kidd’s D-8^5^.

### Regulatory network of anthocyanin biosynthesis in the apple mutant

**Fig. 6:**
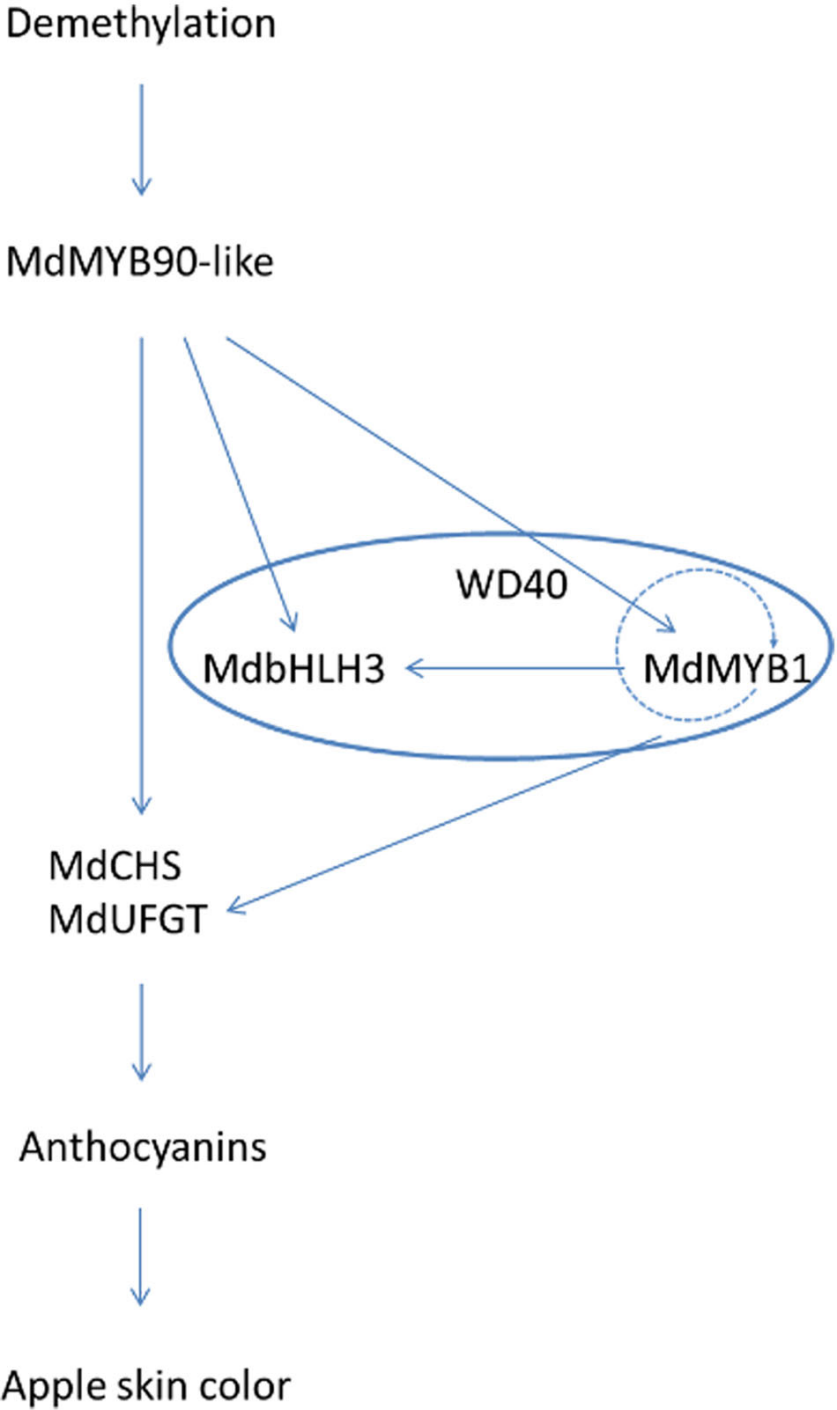
Regulatory network of anthocyanin biosynthesis in the Fuji apple mutant. MdMYB90-like plays a key role in the regulation of anthocyanin biosynthesis. Arrows denote direct activation of downstream genes. The MYB-bHLH3-WD40 regulatory complex was grouped into a cycle. The arrow with a dotted line denotes possible gene activation

The anthocyanin biosynthesis pathway is well known, and the key regulatory genes controlling the pathway have been studied in many plants. Anthocyanin biosynthesis is regulated by both developmental and environmental factors through specific activation or repression of MYB transcription factors. MYB–bHLH–WD40 regulatory complexes are thought to activate specific parts of the pathway by different MYB transcription factors^43^. To date, many MYB transcription factors have been identified. Here, we report the identification of a new MYB gene, *MdMYB90-like*, from an apple mutant. In this mutant, demethylation in two regions of its promoter correlated with increased expression and was probably the cause for its upregulation. MdMYB90-like could bind to cis-elements in other regulatory genes, such as *MdMYB1* and *MdbHLH3*, as well as structural genes in the pathway. Activated MdMYB1 could activate its own expression, as well as structural genes in the pathway, and thus promote the biosynthesis of anthocyanins (Fig. 6). MdMYB90-like played a key role in the regulation of anthocyanin biosynthesis in two possible ways: direct activation of anthocyanin biosynthesis genes (*MdCHS* and *MdUFGT*) and indirect activation of these genes through other transcription factors (*MdMYB1* and *MdbHLH3*).

## Materials and methods

### Plant materials

The Fuji apple (*Malus domestica* Brokh cv. Fuji) and its bud mutant (M_Fuji) were previously observed on a branch at the experimental orchard of Nanjing Agricultural University in Shilaojia County, Jiangsu Province, China^25^. The bud mutant was clonally propagated by grafting, and the mutant phenotype was stably inherited. Flowers on both the wild-type and the mutant were pollinated on April 25, 2016. Young fruits 30 days after pollination (30 DAP) were bagged with two-layer paper bags (Hong Tai, Xi’an, China), which had an inner layer made of red paper with a wax coating and a brown bag as the outside layer. Bags were removed 30 days before harvesting (150 DAP), and samples were collected at five time points: 0, 4, 8, 12, and 16 days after bag removal (DABR) in 2016 for anthocyanin content analysis. Samples of fruits from Fuji (O1, O2, O3) and it’s mutant (M1, M2, M3) were collected at 4, 8, and 12 DABR for RNA extraction and RNA-seq analyses. Nine fruits from each sample were randomly divided into three groups and analyzed as three biological replicates. To speed up anthocyanin accumulation, apples and calli were exposed to continuous light.

### Anthocyanin analysis

Anthocyanin analysis was conducted according to methods reported by Pirie and Mullins^52^ and Xu et al.^34^. Samples (0.2 g) were added to 10 mL precooled hydrochloric acid/methanol (1/99, v/v) solution and extracted in the dark at room temperature for 2 h. All samples were measured in triplicate, and the absorbance at 530 nm and 600 nm was determined by a spectrophotometer. The relative anthocyanin content (*Q*) was calculated as *Q* = OD530-OD600, and *Q* = 0.01 was defined as one unit of anthocyanin content for convenience.

### DNA extraction, RNA extraction, library construction, and RNA-seq

Total RNA was isolated from each sample by using a Mini BEST Plant RNA Extraction kit (Takara Biomedical Technology-Beijing Co., Ltd, China). mRNA was enriched by oligo(dT) beads, and then the enriched mRNA was fragmented randomly into short fragments and reverse transcribed into cDNA with random primers. Second-strand cDNA was synthesized by DNA polymerase I, RNase H, dNTPs, and buffer. Then, the cDNA fragments were purified with a QiaQuick PCR extraction kit, end-repaired, poly(A)-added, and ligated to Illumina sequencing adapters. The ligation products were size selected by agarose gel electrophoresis, PCR-amplified and sequenced using Illumina HiSeqTM 2500 by Gene Denovo Biotechnology Co. (Guangzhou, China).

Genomic DNA (gDNA) was extracted from apple samples by using a Mini BEST Plant DNA Extraction kit (TaKaRa).

### Mapping of reads to the reference genome, gene annotation, and gene expression analysis

By base calling, the original image data produced by the sequencer were transferred into sequences, which were defined as “raw reads”. Clean reads were obtained after the removal of adaptor sequences and reads with >10% unknown bases (N). Differentially expressed genes (DEGs) were selected by comparing the data of different samples according to the reported methods^53^. The threshold for DEGs was a false discovery rate (FDR) ≤0.001, an absolute value of log2 ratio ≥1, and at least one sample read >10. Web Gene Ontology Annotation Plot (WEGO) was used to perform GO classification of the DEGs and to understand the distribution of gene functions in the species at the macro level^54^. Pathway-based analysis was performed by searching the KEGG pathway-related database (https://www.kegg.jp/)^55^. A pathway with a *Q* value ≤0.05 was defined as significantly enriched in differentially expressed genes.

### Gene expression analysis by qRT-PCR

Transcription levels obtained by RNA-seq of 20 selected DEGs were confirmed by qRT-PCR. The selected DEGs were chosen among the three libraries based on their relation to secondary metabolism, flavonoid metabolism, and transcription factors. 18S RNA was used as the reference gene. Gene-specific primers were designed using Primer3 software and are listed in Supplementary Table S5. RNA was extracted from different stages of mutant (M1, M2, and M3) and wild-type apples (O1, O2, and O3). qRT-PCR was performed with an ABI 7300 Real-Time PCR System to analyze gene expression according to the manufacturer’s instructions. All reactions were carried out using SYBR Green Master Mix (SYBR Premix EX TaqTM. TaKaRa) in a total volume of 20 µL, and PCR amplification was performed with the following parameters: 95 °C denaturing for 5 min, followed by 40 cycles of 95 °C denaturing for 5 s, 55 °C annealing for 30 s, and 72 °C extension for 30 s. All reactions and nontemplate controls were performed in triplicate. Relative transcription levels were calculated using the 2-ΔΔCt method^56^. Each measurement was performed with three biological replicates.

### Analysis of the gene sequence and phylogenetic tree construction

DNA fragments of the following gene promoters, *MdCHS* (LOC103443512), *MdUFGT* (LOC103417897), *MdANS* (LOC103437326), *MdBHLH3* (LOC103449015), *MdMYB1* (LOC103444202), and *MdMYB90-like* (HF36881-RA), and the CDSs of *MdMYB90-like* (621 bp) and *MdMYB1* were PCR-amplified from Fuji apple. The primers are listed in Supplementary Table S5. The promoters were sequenced and analyzed using PlantCARE online tools (http://bioinformatics.psb.ugent.be/webtools/plantcare/html/) for cis-acting regulatory elements.

Protein sequences of MdMYB90-like, MdMYB1, and other MYBs (*Arabidopsis* AtMYB113, AtMYB114, AtMYB75, AtMYB90; *Pyrus bretschneideri* PbMYB10, *Fragaria ananasa* FaMYB1, FaMYB10, and *Malus domestica* MdMYB10, MdMYB3, MdMYB9, MdMYBA, MdSIMYB1) were aligned using the clustalw2 program (https://www.ebi.ac.uk/Tools/msa/clustalw2/). The phylogenetic tree was constructed using the MEGA-X program with the neighbor-joining statistical method and bootstrap analysis with 1000 replications.

### Subcellular localization

The PCR-amplified full-length CDSs of *MdMYB90-like* and *MdMYB1* were sequenced and cloned into the pC29_35S:GFP5_his6 vector (Supplementary Fig. S6).

The recombinant plasmids MdMYB90-like-GFP and MdMYB1-GFP were used for transit gene expression analysis. Two-centimeter squares were cut from fresh onion and placed on MS hypertonic media. After dark culture at 25 °C overnight, epidermal cells of the onion’s inner skin were peeled off, placed on MS medium, and transformed by particle bombardment. Transformed tissue was incubated overnight at 25 °C, and GFP expression was detected by LSM 710 NLO laser confocal microscopy (Zeiss, Germany). A 35S:GFP vector was used as a positive control.

For subcellular localization in tobacco leaves, *Agrobacterium* harboring the 35S:GFP, 35S:MdMYB90-like-GFP, and 35S:MdMYB1-GFP constructs were infiltrated and transiently expressed in tobacco leaves. GFP signals were captured under a laser confocal microscope.

### Yeast one-hybrid (Y1H) assay

The PCR fragments of the promoters *MdUFGT*, *MdCHS*, *MdMYB1*, *MdBHLH3*, *MdANS,* and *MdMYB90-like* were inserted into the pLacZi vector (Clontech Laboratories, USA) to generate pLacZi-MdUFGT, pLacZi-MdCHS, pLacZi-MdMYB1, pLacZi-MdBHLH3, pLacZi-MdANS, and pLaczi-MdMYB90-like, respectively (Supplementary Fig. S6). The full-length CDSs of *MdMYB90-like*, and *MdMYB1* were ligated into the pB42AD vector (Clontech) to generate pB42AD-MdMYB90-like and pB42AD-MdMYB1, respectively (Supplementary Fig. S6). The pairs of pLacZi-MdUFGT/pB42AD-MdMYB90-like, pLacZi-MdUFGT/pB42AD-MdMYB1, pLacZi-MdCHS/pB42AD-MdMYB90-like, pLacZi-MdCHS/pB42AD-MdMYB1, pLacZi-MdBHLH3/pB42AD-MdMYB90-like, pLacZi-MdBHLH3/pB42AD-MdMYB1, pLacZi-MdANS/pB42AD-MdMYB90-like, pLacZi-MdANS/pB42AD-MdMYB1, pLacZi-MdMYB1/pB42AD-MdMYB90-like, pLacZi-MdMYB1/pB42AD-MdMYB1, pLaczi-MdMYB90-like/pB42AD-MdMYB90-like, and pLaczi-MdMYB90-like/pB42AD-MdMYB1 were cotransformed into the yeast strain EGY48 according to the published method^57^. The transformed yeast cells were cultured in the dark on SD/-Trp/-Ura medium for 48 h at 30 °C and then placed onto medium containing 5-bromo-4-chloro-3-indolyl-β-d-galactopyranoside (X-gal) for blue color development at 30 °C. Empty pB42AD vectors with pLacZi-MdCHS, pLacZi-MdUFGT, pLacZi-MdANS, pLacZi-MdBHLH3, pLacZi-MdMYB1, and pLacZi-MdMYB90-like were used as negative controls.

### Overexpression of MdMYB90-like in apple calli

The full-length CDS of *MdMYB90-like* was cloned into a pCAMBIA1301 vector^58^ to generate a 35S:MdMYB90-like construct (Supplementary Fig. S6). The recombinant plasmid was introduced into *Agrobacterium* strain LBA4404 and transformed into “Orin” apple calli according to the method described by An et al.^59^. Transgenic calli were screened based on hygromycin resistance. Transgenic calli and WT calli were grown at 24 °C under dark conditions and subcultured every 15 days on media supplemented with hygromycin. Three lines of transgenic calli were harvested, transferred to new plates, and cultured for 5 days under light conditions. Anthocyanin contents and the expression of genes related to anthocyanin biosynthesis were analyzed in both transgenic and WT calli.

### Transient expression of MdMYB90-like in apple skin

35S:MdMYB90-like was cloned into the pGreenII62-SK vector to generate 62SK-MdMYB90-like (Supplementary Fig. S6). The empty pGreenII62-SK vector was used as a negative control. The vectors were introduced into GV3101(p-soup). Cultured *A. tumefaciens* cells were injected into Fuji and Golden Delicious fruit skins, and the infiltrated fruits were cultured at room temperature under continuous light conditions.

### Dual-luciferase assay

The 62SK-MdMYB90-like effectors (35S:MdMYB90-like cloned into pGreenII62-SK vector) and reporter constructs (the promoter fragments of MdMYB1, MdbHLH3, MdCHS, and MdUFGT cloned into the pGreenII 0800-LUC vectors, Supplementary Fig. S6) were transformed into *A. tumefaciens* GV3101(p-soup). The bacteria were mixed and coinjected into tobacco leaves and cultured for 2 days under light conditions. A living fluorescence imager was used to observe the fluorescence of the tobacco leaves, which were also sampled to measure LUC/REN activity.

### Electrophoretic mobility shift assay

Electrophoretic mobility shift assay (EMSA) was performed using an EMSA Probe Biotin Labeling Kit and a Chemiluminescent EMSA Kit (Beyotime Biotechnology, Shang Hai, China). The *MdMYB90-like* gene was cloned into the pMAL-c5X vector (Supplementary Fig. S6), which was then transformed into Rosetta (DE3) cells for the subsequent production of the MdMYB90-like-MBP fusion protein. Probes specific for the promoter fragments and their mutants (Supplementary Table S6) were synthesized by Sangon Biotechnology Co., Ltd. (Shanghai, China). In BHLH3, the 5′-TAACCA-3′ motif was replaced by 5′-TGGTAA-3′ in the mutant probe; in CHS and UFGT, the 5′-CAACTG-3′ motif was replaced by 5′-TGGTAA-3′ in the mutant probe; in MYB1, the 5′-CAACGG-3′ motif was replaced by 5′-TGGTAA-3′ in the mutant probe.

Binding reactions contained 2 µL of 1× binding buffer, 1 µL of MYB90-like-MBP protein extract, 1 µL of biotin-labeled probes, and 1 µL of unlabeled competitors or mutant probes in a total volume of 10 µL. Reactions were electrophoresed, transferred, and detected as described in the Chemiluminescent EMSA Kit (Beyotime Biotechnology).

### DNA methylation analysis

Genomic DNA (1 µg) from apple skins of both Fuji and its colored mutant was digested separately with the methylation-specific endonuclease enzyme McrBC (New England Biolabs) in a 100 µl total volume including 1 µg DNA, 1× NEB2 buffer, 1× BSA (bovine serum albumin), 20 U McrBC and 1 mM GTP or ddH_2_O (as a negative control). The reaction was performed at 37 °C overnight and was stopped by heating at 65 °C for 20 min. The digested gDNA was used as a template for PCR analysis. Primers were designed to divide the promoter and exon sequences of both *MdMYB1* and *MdMYB90-like* into 14 fragments (Supplementary Table S5). PCRs were performed with the following parameters: 95 °C denaturing for 1 min, followed by 35 cycles of denaturing at 95 °C for 30 s, annealing at 55 °C for 30 s and extension at 72 °C for 1 min, and a final 5 min extension at 72 °C. PCR amplification products were checked by agarose gel (1.2%) electrophoresis.

To analyze methylated nucleotides in the promoter region of *MdMYB90-like*, bisulfite sequencing was performed. Genomic DNA (1 µg) from apple skins of both Fuji and its colored mutant was treated with a DNA Methylation Kit (www.cwbiotech.com). Treated gDNA and untreated gDNA (control) were used as templates to amplify two regions of the *MdMYB90-like* gene promoter (−1183 to −988 and −2018 to −1778), which showed different levels of methylation between the wild-type and the mutant. The primers are listed in Supplementary Table S5. Amplified PCR products were cloned, sequenced, and analyzed using cytosine methylation analysis online tools CyMATE (http://www.cymate.org/). Methylation levels as the percentage of methylated nucleotides were calculated from nine independent clones.

### Statistical analysis

For statistical analysis, three replicates were performed. Statistical analysis was performed using Microsoft Excel 2010. Each value represents the mean ± SE of three independent biological replicates. The differences between data were analyzed with *t* tests. A *P* value <0.05 was considered statistically significant.

## Supplementary information

Supplementary figures

Quality assessment of RNA sequencing by Illumina HiSeqTM 2500

DEGs in O1-vs-M1 group samples

DEGs in O2-vs-M2 group samples

DEGs in O3-vs-M3 group samples

List of primers and probes

## Data Availability

The datasets used and/or analyzed during this study are available from the corresponding author on reasonable request. All sequence data were deposited in GenBank under SRA accession number PRJNA549998.
