# Supplementary material for: The R2R3-type MYB transcription factor MdMYB90-like is responsible for the enhanced skin color of an apple bud sport mutant
Source: Hortic Res. 2021 Jul 1;8:156. doi: 10.1038/s41438-021-00590-3 (PMC8245648; doi:10.1038/s41438-021-00590-3)
Supplement: Supplementary file 1 — Supplementary figures [file 41438_2021_590_MOESM1_ESM.docx]

**Supplementary figures:**

**Figure S1.**


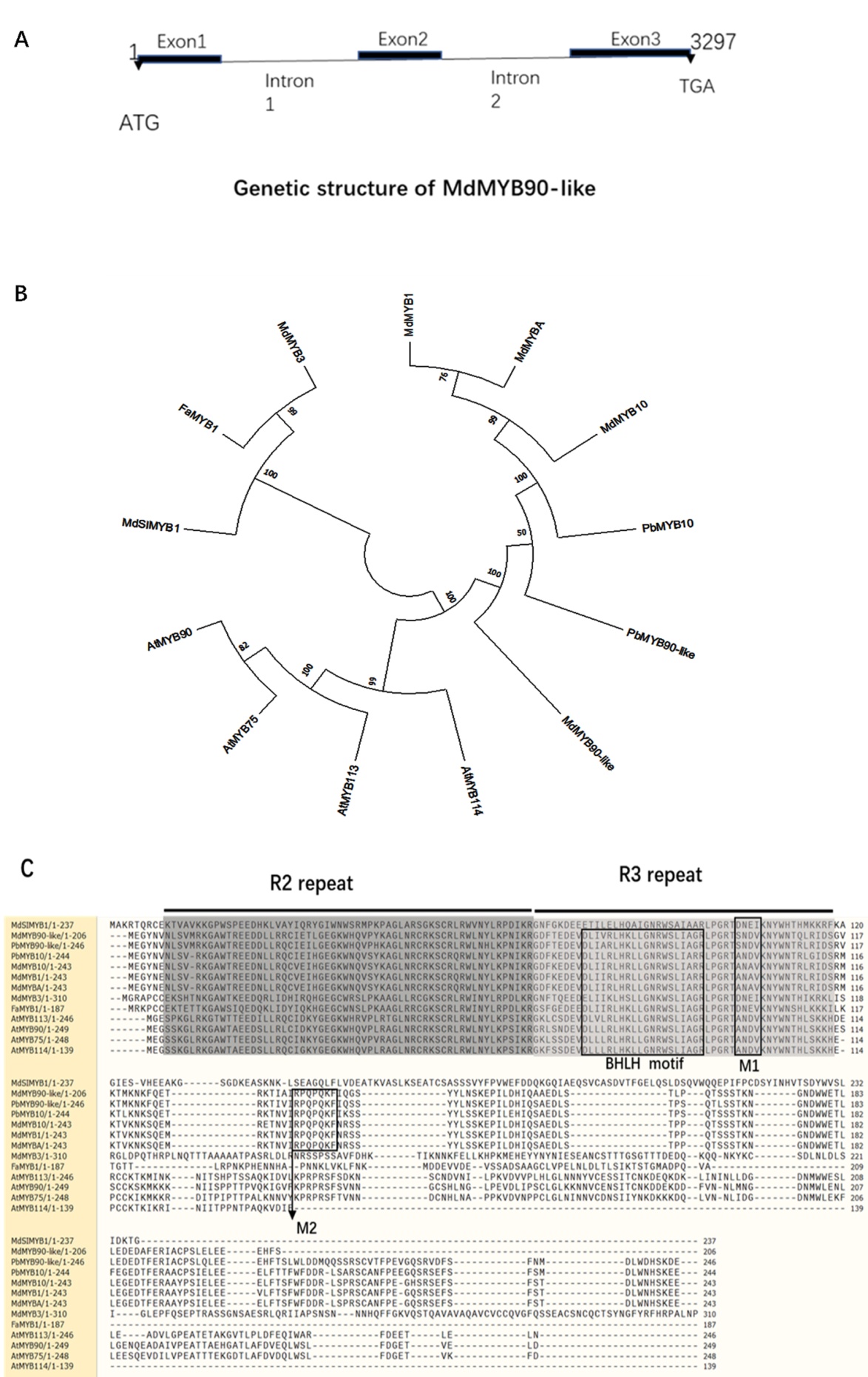


**Supplementary Figure S1.** **Sequence analysis of MdMYB90-like.** A) Genome structure of *MdMYB90-like* gene; B) Phylogenetic analysis of R2R3 MYBs from different species, including AtMYB113, AtMYB114, AtMYB75, and AtMYB90 from Arabidopsis; PbMYB10 from *Pyrus bretschneideri*, FaMYB1 from *Fragaria ananasa*, and MdMYB10, MdMYB3, MdMYBA, MdSIMYB1, MdMYB1 and MdMYB90-like from *Malus domestica*; C) Sequences alignment of MdMYB90-like protein with other MYB transcription factors. Black bars on top of the sequences indicated the R2 and R3 domains, M1 (NEDI/ANDV), M2 ([K/R] Pxxx[K/T] [F/Y]), and bHLH motifs were boxed.

**Figure S2.**

**
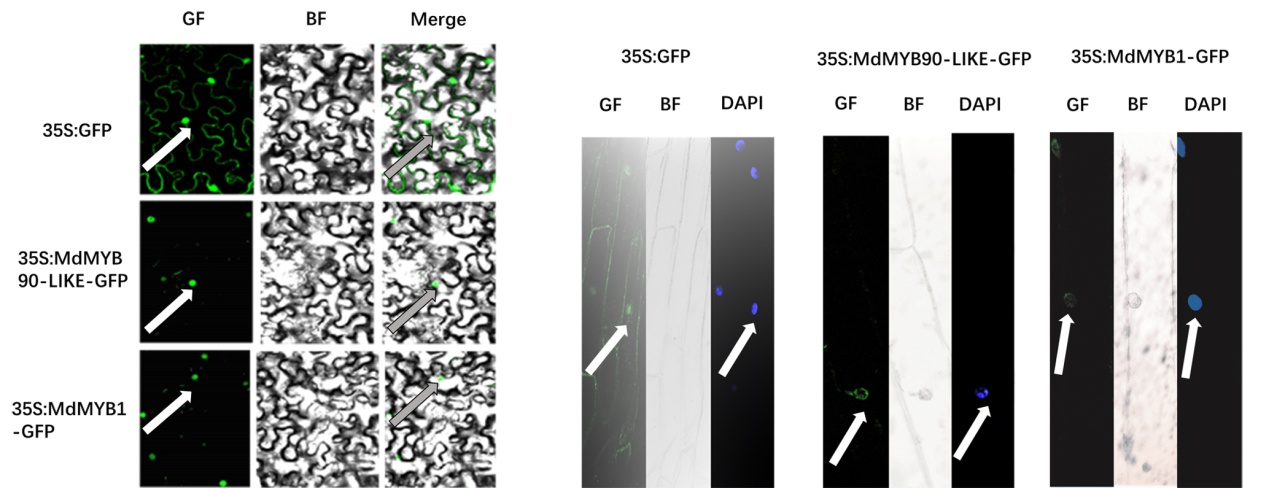
**

**Supplementary Figure S2. Subcellular localization of MdMYB90-like.** A) 35S:GFP (top panel), 35S:MdMYB90-like-GFP (middle panel), and 35S:MdMYB1-GFP (bottom panel) constructs were transiently expressed in the tobacco leaves. B) 35S:GFP (left panel), 35S:MdMYB90-like-GFP (middle panel), and 35S:MdMYB1-GFP (right panel) constructs were transiently expressed in onion epidermal cells. GFP signals were captured under laser confocal microscope (left panels). Both MdMYB1-GFP and MdMYB90-like-GFP were localized in the tobacco and onion nucleus, while free GFP was localized in both nucleus and cytosol. Arrows denote the nuclei.

**Figure S3.**


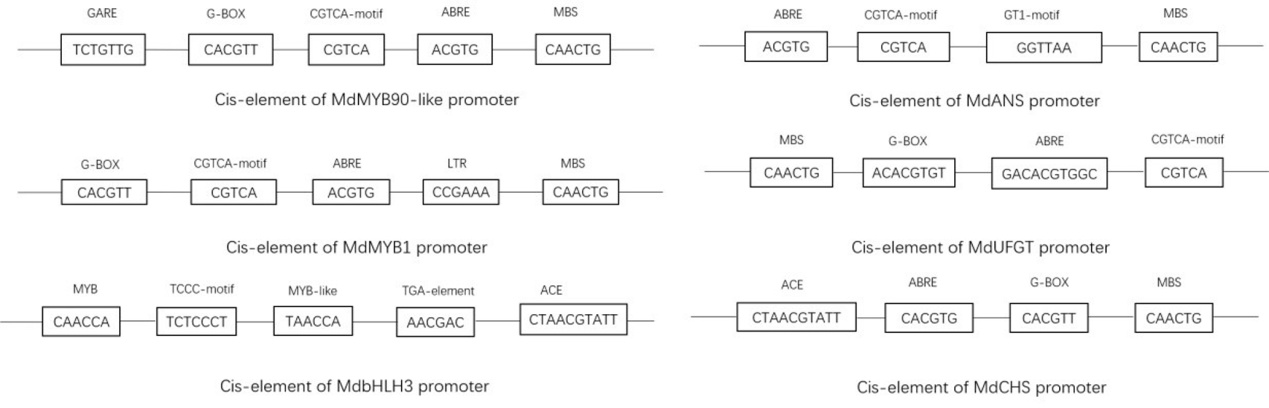


**Supplementary Figure S3. Cis-acting regulatory elements in gene promoters**. Cis-acting regulatory elements were analyzed by searching the PlantCARE database of (http://bioinf ormatics.psb.ugent.be/w ebtools/plantcare/html/) in the promoters of MdMYB90-like, MdMYB1, MdBHLH3, MdANS, MdUFGT and MdCHS. G-BOX: cis-acting regulatory element involved in light responsiveness; ABRE: cis-acting element involved in the abscisic acid responsiveness; GT1-motif: light responsive element; TGA:auxin response element; CGTCA-motif: cis-acting regulatory element involved in the MeJA-responsiveness; LTR: cis-acting element involved in low-temperature responsiveness; MBS: MYB-binding site; TCCC-motif: part of a light responsive element; GARE motif: gibberellin-responsive element; ACE: cis-acting element involved in light responsiveness, MSA-like: cis-acting element involved in cell cycle regulation.

**Figure S4.**

**
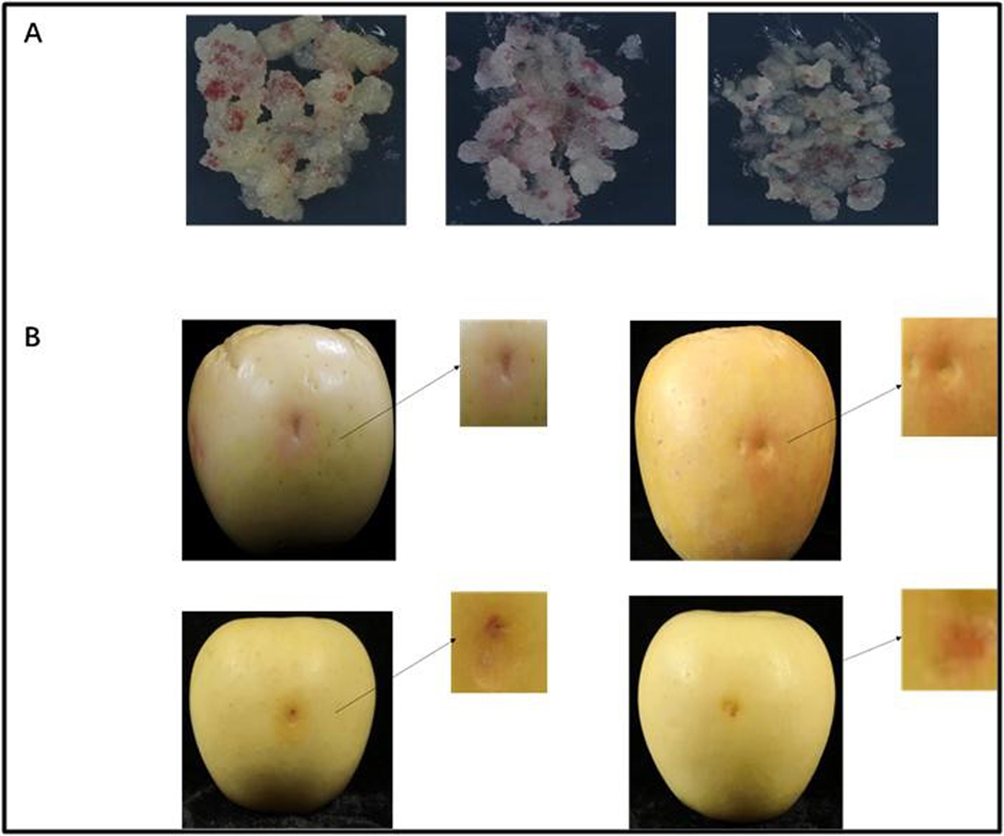
**

**Supplementary Fig. S4**. Anthocyanin accumulation in different transgenic apple calli lines (A) and additional agroinfiltrated apples (B).

**Figure S5.**

**
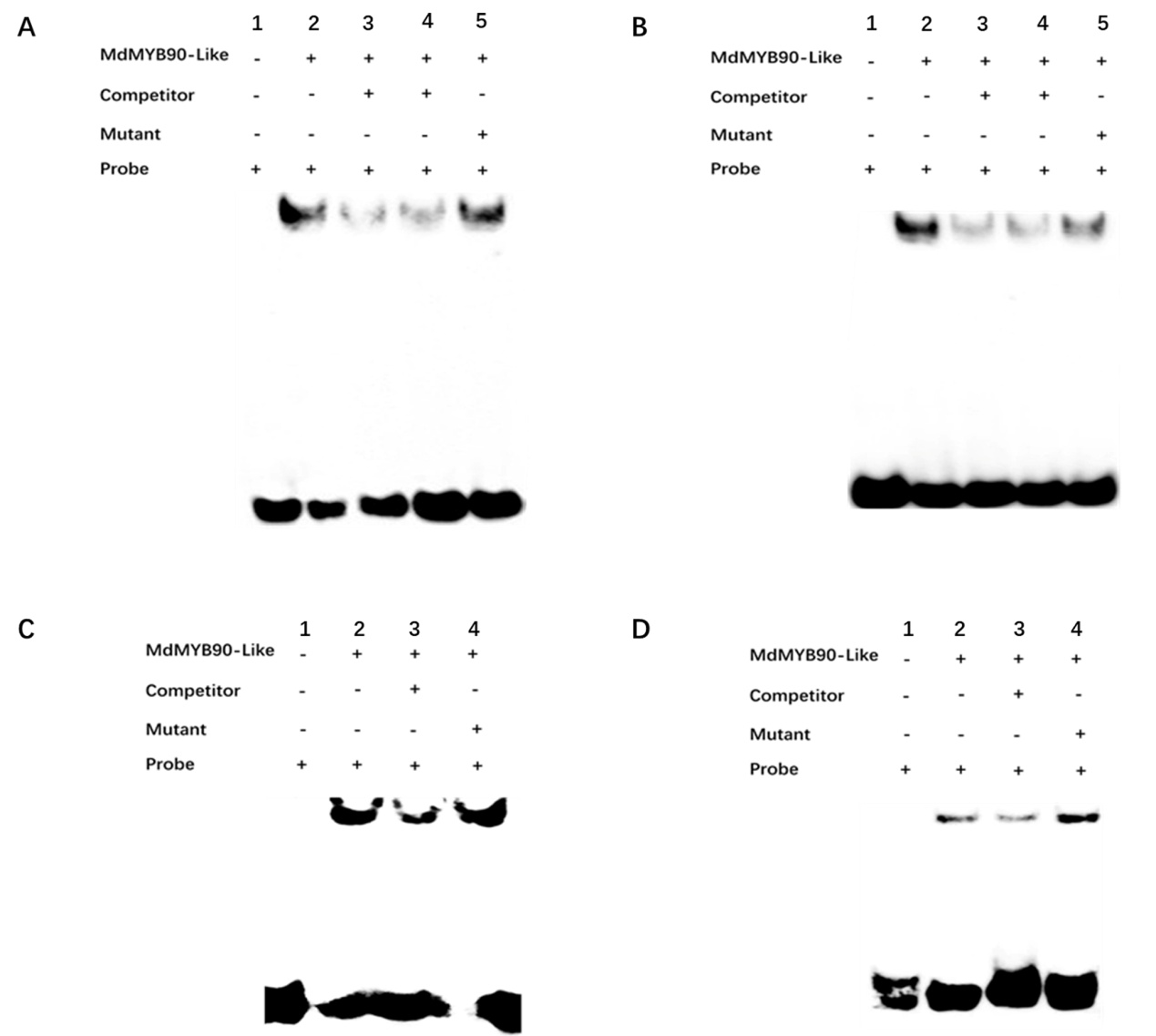
**

**Supplementary Figure S5.** **Electrophoretic mobility shift assay.** MdMYB90-like-MBP fusion protein directly bounds to the MYB-binding elements of the MdCHS (A), MdUFGT (B) MdBHLH3 (C) and MdMYB1(D) promoters. Lane 1, probes without MdMYB90-like-MBP fusion protein; lane 2, probes with MdMYB90-like-MBP fusion protein; lane 3 and 4 in (A) and (B), lane 3 in (C) and (D), probes with MdMYB90-like-MBP fusion protein and competitor probes; lane 5 in (A) and (B), lane 4 in (C) and (D), probes with MdMYB90-like-MBP fusion protein and mutant probes.

**Figure S6.**

**A)**


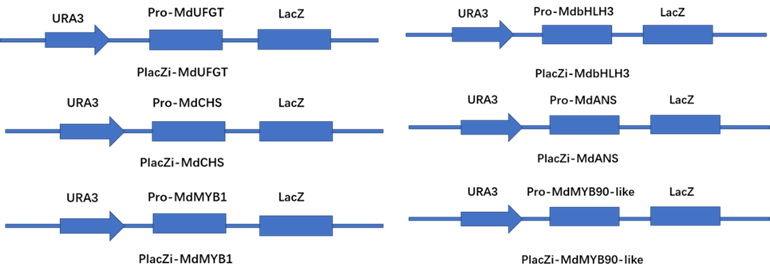


**B)**


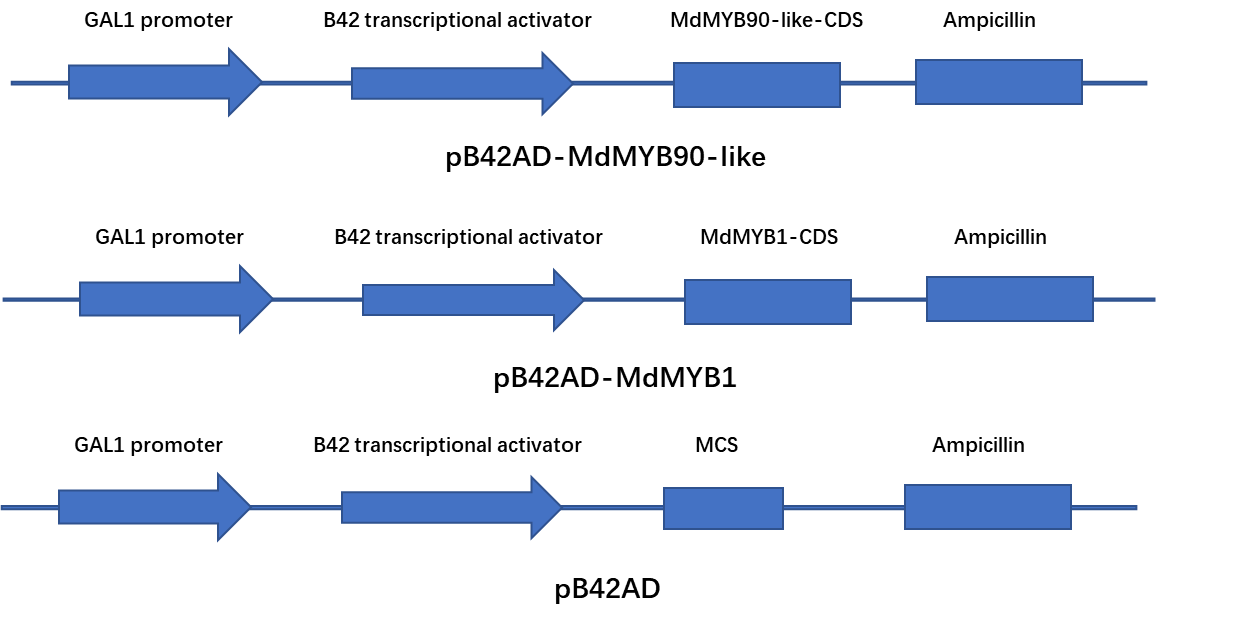


**C)**


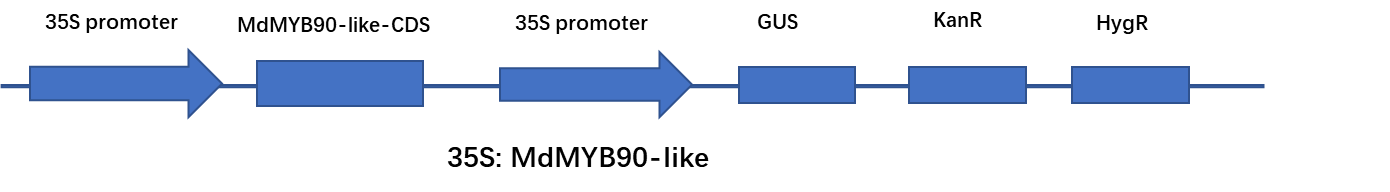


**D)**


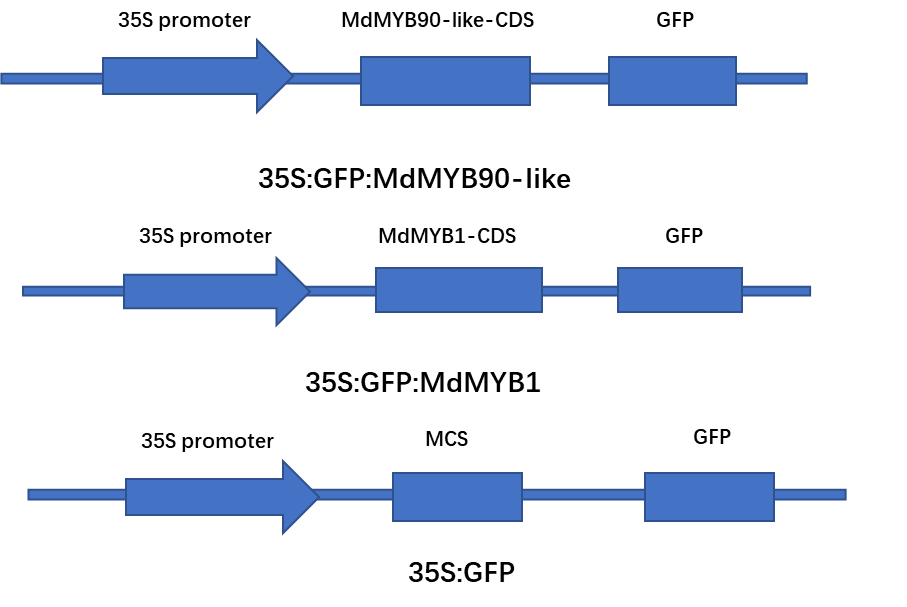


**E)**


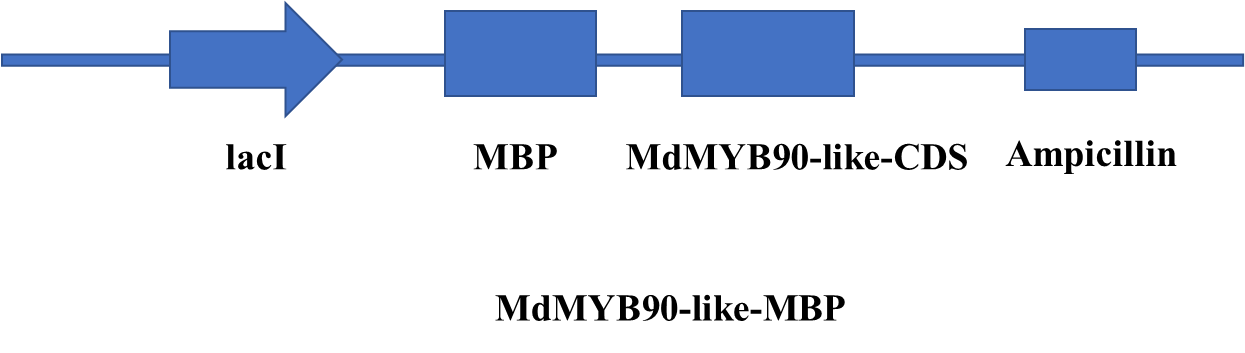


**Figure S6. Diagrams of constructs.** A) The PCR fragments of the following promoters: MdUFGT, MdCHS, MdMYB1, MdBHLH3, MdANS, MdMYB90-like were inserted into the pLacZi vector (Clontech) to generate pLacZi-MdUFGT, pLacZi-MdCHS, pLacZi-MdMYB1, pLacZi-MdBHLH3, pLacZi-MdANS, pLaczi-MdMYB90 respectively. B) The full-length coding sequence (CDS) of MdMYB90-like and MdMYB1 were ligated into pB42AD vector (Clontech) to generate pB42AD-MdMYB90-like and pB42AD-MdMYB1, respectively. C) The full-length coding sequencing (CDS) of MdMYB90-like was cloned into a pCAMBIA1301 vector to generate 35S: MdMYB90-like construct. D) The PCR amplified full-length coding sequences (CDS) of *MdMYB90-like* and *MdMYB1* were sequenced and cloned to pC29_35S:GFP5_his6 vector. E) The full-length coding sequencing (CDS) of MdMYB90-like was cloned into a pMAL-c5X vector to generate MdMYB90-like-MBP construct.
