# Supplementary material for: The R2R3-type MYB transcription factor MdMYB90-like is responsible for the enhanced skin color of an apple bud sport mutant
Source: Hortic Res. 2021 Jul 1;8:156. doi: 10.1038/s41438-021-00590-3 (PMC8245648; doi:10.1038/s41438-021-00590-3)
Supplement: Supplementary file 2 — Quality assessment of RNA sequencing by Illumina HiSeqTM 2500 [file 41438_2021_590_MOESM2_ESM.docx]

| Sample | Clean Reads Num | HQ clean Reads Data(bp) | HQ Clean Data(bp) | Q20(%) | Q30(%) | N(%) | GC(%) |
| --- | --- | --- | --- | --- | --- | --- | --- |
| M1 | 22535156 | 22323786 (99.06%) | 3344845746 | 3217054903 (96.18%) | 3032365059 (90.66%) | 26186 (0.00%) | 1660229737 (49.64%) |
| O1 | 25363188 | 25158118 (99.19%) | 3769373822 | 3632452224 (96.37%) | 3435384503 (91.14%) | 29715 (0.00%) | 1855007289 (49.21%) |
| M2 | 22099366 | 21930960 (99.24%) | 3285525419 | 3166070571 (96.36%) | 2995183930 (91.16%) | 26315 (0.00%) | 1607436250 (48.92%) |
| O2 | 27419664 | 26982800 (98.41%) | 4040732065 | 3905393601 (96.65%) | 3711969147 (91.86%) | 32277 (0.00%) | 1991777679 (49.29%) |
| M3 | 23440962 | 23294704 (99.38%) | 3489762456 | 3373859211 (96.68%) | 3205853328 (91.86%) | 27137 (0.00%) | 1675778714 (48.02%) |
| O3 | 20209566 | 20058590 (99.25%) | 3005007145 | 2896210191 (96.38%) | 2742388170 (91.26%) | 23646 (0.00%) | 1453937870 (48.38%) |

Table S1. Quality assessment of RNA sequencing by Illumina HiSeqTM 2500
