# Supplementary material for: The R2R3-type MYB transcription factor MdMYB90-like is responsible for the enhanced skin color of an apple bud sport mutant
Source: Hortic Res. 2021 Jul 1;8:156. doi: 10.1038/s41438-021-00590-3 (PMC8245648; doi:10.1038/s41438-021-00590-3)
Supplement: Supplementary file 6 — List of primers and probes [file 41438_2021_590_MOESM6_ESM.docx]

**Table S5. List of primers and probes**

1. **qRT-PCR primer**

| Gene name | Gene bank ID | Primer sequence (5'-3') |
| --- | --- | --- |
| ANS | LOC103437327 | F: TGCCAATGACCAGGCCTCTG |
|  |  | R: CAATCCCAAGCCAAGTGACA |
| DMR6 | LOC103444963 | F: AAATATTGAATGGAATATCG |
|  |  | R: GTAGTAGTTCGCTGCAAGAAT |
| PAL-1 | LOC103430265 | F: ATGGAACCAGGAAATAACC |
|  |  | R: CATTGCTTCAGCTGCCATCC |
| PAL-1like | LOC103433222 | F: AACCATCACGCAAAATGGTCA |
|  |  | R: TAGACCCCAGTTCAATGGGT |
| UFGT | LOC103455522 | F: ATGAGCTCAAAGCCACATGCT |
|  |  | R: GTAGGAGATGGGATGGCAAA |
| UFGT | LOC103420802 | F: AATCGAGCCACAAACAACTA |
|  |  | R: GCAGCTAGGCGGCGAACGAT |
| 4CL | LOC103426517 | F: ATGACCATTGCTTCCAGTTCC |
|  |  | R: TTTGTTGAGAGGTAGAATTAA |
| ANS | LOC103437326 | F: GGAGAAGATCATCCTTAAG |
|  |  | R: TCACTTGGGGAGCAAAGCCT |
| DFR | LOC103448549 | F: AAGGTTCCATCAAGCATTCA |
|  |  | R: AAAGATGTGGGCATTGCATA |
| MYB1 | LOC103444202 | F: TCTCCCTTTCTCTAGCCACGA |
|  |  | R: ACGTGCTCTGGCATCCTTATC |
| UFGT | LOC103440008 | F: GGACCAAGTAGAGGCCAAGT |
|  |  | R: TCCTCCACCATCCTTGCATT |
| MYB90-like | TCONS_00045044 | F: CCACATTCAAGCAGCAGAAG |
|  |  | R: GCTGGGACATGCAATTCTTTC |
| CHS | LOC103443512 | F: CAAGCGCCTCATGATGTACC |
|  |  | R: GTTGTTTTCGGCCAAGTCCT |
| CHS | LOC103443513 | F: ATGGTTACAGTCGAGGAAG |
|  |  | R: ACCTTGTGCTCGCTGTTG |
| CHI | LOC103430446 | F: ATGGGCACTGAAGTTGTAAT |
|  |  | R: GTGTGACACAACTTCAGGGT |
| F3'H | LOC103437875 | F: ATGTTTGTTCTCATATTCTTCA |
|  |  | R: CCTAAGTGGGGCAAGTTTC |
| 4CL | LOC103447296 | F: ATGGAGAAATCTGGGTTCG |
|  |  | R: CGGAGGACTCGCCGTCGATG |
| FLS | LOC103413102 | F: GAAGCTAATGAAGAGTATGC |
|  |  | R: GACATGGTGGGTAGTAGTTA |
| F3H | LOC103450635 | F: ATGGAAGAAATCAATCCATC |
|  |  | R: CTTAGCGGCAAGCTCTTCCAC |
| ANR  SIMYB1  BHLH3 | LOC103413696  LOC103412495  LOC103449015 | F: ATGCCTGAGAGCATGGGAGT  R: CCCAGTGAGATGCTATTTCTG  F: ATGGCGAAAAGGACTCAGCG  R: GGCATCCGACTCCAATTCC  F: GTAAAGAGTTGCGAAGTGGG  R: GTAAAGGTTCTGGCTGAGGT |
| 18sRNA |  | F: ACACGGGGAGGTAGTGACAA |
|  |  | R: CCTCCAATGGATCCTCGTTA |

1. **CDS (coding sequence) primers**

MdMYB90-like-CDS-F: ATGGAGGGATATAACGTTAACTTG

MdMYB90-like-CDS-R: TCAACTGAAGTGTTCTTCCTCT

MdMYB1-CDS-F: ATGGAGGGATATAACGAAAAC

MdMYB1-CDS-R: CTATTCTTCTTTTGAATGATTCCA

1. **Promoter primers**

MdMYB90-like-pro-F: GTAATGTCGAGCACTTCAAGAA

MdMYB90-like-pro-R: CCTCCATCTCTTATCTGCTAGCTAG

MdMYB-1-pro-F: CGTTCGAAGGTCTAAGGTGACATA

MdMYB-1-pro-R: CATCTCTTATCTGCTAGCAGCTAAGC

MdANS-pro-F: GAACATATGTGGACTAATCCAACCAAAA

MdANS-pro-R: CACCATTTTTGGAGCTGGCTTTCGACA

MdUFGT-pro-F: CTGACTTCTAGAGTGAGTATGTCAGAATA

MdUFGT-pro-R: GGGCAGCGGCGCTGCCATTACAGCTTACA

MdBHLH3-pro-F: GGATGTGGAAATATTGGTAG

MdBHLH3-pro-R: AACTGAGGCCTGCAACATAC

MdCHS-pro-F: AACCTTTGGAGGCTGCGTTTGGGAAGG

MdCHS-pro-R: CACCATTTGTTTTTATATCCGATCGTCG

1. **MdMYB90-ovexpressed primers**

MYB90-OE-F: GGGGTACCATGGAGGGATATAACGTTAACTTG

MYB90-OE-R: GCTCTAGATCAACTGAAGTGTTCTTCCTCT

pG62SK-MYB90-F: TCCCCCGGGCTGCAGGAATTCATGGAGGGATATAACGTTA

pG62SK-MYB90-R: GATAAGCTTGATATCGAATTCTCAACTGAAGTGTTCTTCCT

1. **Subcellular localization primers**

MdMYB90-F: TCGACGATAGCCATGCTCGAGATGGAGGGATATAACGTTAACTTG

MdMYB90-R: TTCTTCTCCTTTACTAAGCTTTCAACTGAAGTGTTCTTCCTCT

MdMYB1-F: TCGACGATAGCCATGCTCGAGATGGAGGGATATAACGAAAAC

MdMYB1-R: TTCTTCTCCTTTACTAAGCTTCTATTCTTCTTTTGAATGATTCCA

1. **Y1H primers**

plscz-ANS-ProF: GGGGTACCACAGTAAATACATTTAAAACGTGTAAGTT

placz-ANS-ProR: CCCTCGAGTGCTGCTAGCTACCACTAGCAAGA

pLaczi-UFGT-F: GGGGTACCTACAAGGCTAATTAGAAAAGGAG

pLaczi-UFGT-R: CCCTCGAGTCCCTAGATTGACTGTTGGCCT

pLaczi-CHS-F: GGGGTACCATCTCTTTTCCCTTTCCTTTGC

pLaczi-CHS-R: CCCTCGAGAGATGAGGGTCACGTGATTGA

pLaczi-BHLH3-F: GGGGTACCGGATGTGGAAATATTGGTAG

pLaczi-BHLH3-R: CCCTCGAGAACTGAGGCCTGCAACATAC

Placzi-MYB1-F: GGGGTACCAGATCATATATCACGTCACT

Placzi-MYB1-R: CCCTCGAGCTATGCGTATTCTAAAGATGGA

pLaczi-MYB90-1F: GGGGTACCACTGAGGGGAGAATTATGG

pLaczi-MYB90-1R: CCCTCGAGTAGAGTGTCTGCCATAACG

pB42AD-MYB90-F: TGCCTCTCCCGAATTCATGGAGGGATATAACGTTAAC

pB42AD-MYB90-R: CGAGTCGGCCGAATTCTCAACTGAAGTGTTCTTCCTC

pB42AD-MYB1-F: TGCCTCTCCCGAATTCATGGAGGGATATAACGAAAA

pB42AD-MYB1-R: CGAGTCGGCCGAATTCCTATTCTTCTTTTGAATGATTCC

1. **McrBC-analysis primers**
2. **MdMYB1 Primers**

| +1 to +130-F: | ATGGAGGGATATAACGAAAACCTG |
| --- | --- |
| +1 to +131-R: | TACCTGCTTTGTATGAAACTTGG |
| +145 to +257-F: | AGAGCTGCAGACAAAGATGGTTAAA |
| +145 to +257-R: | CCTGTTTCCCAAAAGCCTGTGAAGT |
| +393 to +715-F: | GATAAGACCTCAGCCCCAAAAG |
| +393 to +715-R: | CCAAAGGTCCGTGCTAAAGG |
| -169 to -1-F: | TGCACGTCACTGGCCTTGTA |
| -169 to -1-R: | CTCTTATCTGCTAGCAGCTAA |
| -304 to -168-F: | GTCGTGCAGAAATGTTAGCTTT |
| -304 to -168-R: | ATGTCTGATATCCACAGAAGC |
| -466 to -312-F: | TGAAAAAGCAGCGAAAGCATG |
| -466 to -312-R: | AAGGGAAATCAATCCCAGGGCAT |
| -544 to -450-F: | AGGAGAGAATCCTACTCCATA |
| -544 to -450-R: | CTTTCGCTGCTTTTTCAAGTG |
| -705 to -553-F: | ACGGGCTAGGATTTTCTCCTCTT |
| -705 to -553-R: | CTCTTCTTCATTCCCCTCCTAT |
| -992 to -651-F: | AGGCTGAACCACCTATGAAAAT |
| -992 to -651-R: | TGAGAGGAATGGATGGAATGG |
| -1098 to -988-F: | GCGTGGTCCCGCAAGACAGATAA |
| -1098 to -988-R: | AGCCTTGTTAATTTAACTCCATGTGG |
| -1413 to -1226-F: | ACAACCTTCACAAGGGTTGTCG |
| -1413 to -1226-R: | GTCTTCGTTGGATTCCGTTAAGCG |
| -1706 to -1423-F: | TAAATTTTTTCTAGGGCATAATTTGCC |
| -1706 to -1423-R: | GTGACGTGATATATGATCTTGATGG |
| -1880 to -1678-F: | GTTTGCTGTTGCCATTTTTGAAC |
| -1880 to -1678-R: | TTCCCACGTGTTCAGGGTCCTTT |
| -2024 to -1871-F: | CGTTCGAAGGTCTAAGGTGACATA |
| -2024 to -1871-R: | CAACAGCAAACACCCAAAATCC |

**(2) MdMYB90-like Primers**

| -1 to -170-F: | GCACGTCAGTGGCCGTATAAG |
| --- | --- |
| -1 to -170-R: | CTCTTATCTGCTAGCTAGCAT |
| -374 to -167-F: | GAACAAGCTGGGGTAAGTTTAGA |
| -374 to -167-R: | TGCATGTCTGGATATCCACAG |
| -581 to -375-F: | CAATAAATATTGAACAATCACT |
| -581 to -375-R: | CAACCCTTGGGCTAAATATGA |
| -823 to -533-F: | CTGGGTTAAAACCTAAATTT |
| -823 to -533-R: | GAATCTAAATATTTTTAAGT |
| -987 to -794-F: | CGGTTAGTGATTTTTCAATA |
| -987 to -794-R: | CAACTTTTTTGTTTCAATCCT |
| -1183 to -988-F: | CTGACCCACCATATATGTATA |
| -1183 to -989-R: | ACACCATGAAACTTGTGTAAC |
| -1399 to -1181-F: | TAGGTTAAAACCTAAAATT |
| -1399 to -1182-R: | CAGCCCCTCTAACCCTAAGG |
| -1571 to -1381-F: | TAGCTATATAATTTTATGCCT |
| -1571 to -1381-R: | CTAAAGGTTGGAGATGGT |
| -1792 to -1525-F: | GTCCACTTCTTAGACTTGAA |
| -1792 to -1525-R: | CTATAATATAAAATTTTGTAGC |
| -2018 to -1778F: | GTAATGTCGAGCACTTCAAGA |
| -2018 to -1778R: | AGAAGTGGACAACACAAAATGG |
| +1 to +130-F: | ATGGAGGGATATAACGTTAA |
| +1 to +130-R: | CTTCATCCTCTGTAAAGTCT |
| +131 to +261-F: | TAGATCTTATAGTTAGACTTCA |
| +131 to +261-R: | CTGTTCAAGTAATATGAACCT |
| +263 to +449-F: | GTGGTCATTGATTGCTGGAA |
| +263 to +450-R: | CTGTTCAAGTAATATGAACCTTGGA |
| +447 to +621-F: | GTAAAGAACCAATTCTGGAC |
| +447 to +621-R: | ACTGAAGTGTTCTTCCTCTAAC |

1. **Bisulfite sequencing primers**

-2018 to -1778F: GTAATGTCGAGCACTTCAAGA

-2018 to -1778R: AGAAGTGGACAACACAAAATGG

-1183 to -988-F : CTGACCCACCATATATGTATA

-1183 to -988-R: ACACCATGAAACTTGTGTAAC

1. **Luciferase assay Primers**

pG62SK-MYB90-F: TCCCCCGGGCTGCAGGAATTCATGGAGGGATATAACGTTA

pG62SK-MYB90-R: GATAAGCTTGATATCGAATTCTCAACTGAAGTGTTCTTCCT

pG0800-UFGT-F: ctatagggcgaattgggtaccTACAAGGCTAATTAGAAAAGGAG

pG0800-UFGT-R: caggaattcgatatcaagcttTCCCTAGATTGACTGTTGGCCT

pG0800-CHS-F: ctatagggcgaattgggtaccATCTCTTTTCCCTTTCCTTTGC

pG0800-CHS-R: caggaattcgatatcaagcttAGATGAGGGTCACGTGATTGA

pG0800-bHLH3-F: ctatagggcgaattgggtaccGGATGTGGAAATATTGGTAG

G0800-bHLH3-R: caggaattcgatatcaagcttAACTGAGGCCTGCAACATAC

pG0800-MYB1-F: ctatagggcgaattgggtaccAGATCATATATCACGTCACT

pG0800-MYB1-R: caggaattcgatatcaagcttCTATGCGTATTCTAAAGATGGA

1. **EMSA probes**

BHLH3-F: CCTGCTCAC**TAACCA**ATCAAACCCA

BHLH3-R: TGGGTTTGATTGGTTAGTGAGCAGG

BHLH3m-F: CCTGCTCACTGGTAAATCAAACCCA

BHLH3m-R: TGGGTTTGATTTACCAGTGAGCAGG

UFGT-F: TGGTACAAA**CAACTG**CAATAAAATA

UFGT-R: TATTTTATTGCAGTTGTTTGTACCA

UFGTm-F: TGGTACAAATGGTAACAATAAAATA

UFGTm-R: TATTTTATTGTTACCATTTGTACCA

CHS-F: GACTAATTT**CAACTG**CTCCCAAAGT

CHS-R: ACTTTGGGAGCAGTTGAAATTAGTC

CHSm-F: GACTAATTTTGGTAACTCCCAAAGT

CHSm-R: ACTTTGGGAGTTACCAAAATTAGTC

MYB1-F:  CGGACCACT**CAACGG**AACAAATAAG

MYB1-R: CTTATTTGTTCCGTTGAGTGGTCCG

MYB1m-F: CGGACCACTTGGTAAAACAAATAAG

MYB1m-R: CTTATTTGTTTTACCAAGTGGTCCG
